# Functional genomic analysis delineates regulatory mechanisms of GWAS-identified bipolar disorder risk variants

**DOI:** 10.1186/s13073-022-01057-3

**Published:** 2022-05-20

**Authors:** Rui Chen, Zhihui Yang, Jiewei Liu, Xin Cai, Yongxia Huo, Zhijun Zhang, Ming Li, Hong Chang, Xiong-Jian Luo

**Affiliations:** 1 Key Laboratory of Animal Models and Human Disease Mechanisms of the Chinese Academy of Sciences & Yunnan Province, Kunming Institute of Zoology, Chinese Academy of Sciences, Kunming, Yunnan 650223 China; 2 Kunming College of Life Science, University of Chinese Academy of Sciences, Kunming, Yunnan 650204 China; 3 Department of Neurology, Affiliated Zhongda Hospital, Southeast University, Nanjing, Jiangsu 210096 China; 4 Key Laboratory of Developmental Genes and Human Disease of Ministry of Education, Southeast University, Nanjing, Jiangsu 210096 China; 5 Zhongda Hospital, School of Life Sciences and Technology, Advanced Institute for Life and Health, Southeast University, Nanjing, Jiangsu 210096 China

**Keywords:** Bipolar disorder, Genome-wide association study (GWAS), Functional genomics, Transcription factor–disrupting SNPs, Expression quantitative trait loci (eQTL), Regulatory mechanisms

## Abstract

**Background:**

Genome-wide association studies (GWASs) have identified multiple risk loci for bipolar disorder (BD). However, pinpointing functional (or causal) variants in the reported risk loci and elucidating their regulatory mechanisms remain challenging.

**Methods:**

We first integrated chromatin immunoprecipitation sequencing (ChIP-Seq) data from human brain tissues (or neuronal cell lines) and position weight matrix (PWM) data to identify functional single-nucleotide polymorphisms (SNPs). Then, we verified the regulatory effects of these transcription factor (TF) binding–disrupting SNPs (hereafter referred to as “functional SNPs”) through a series of experiments, including reporter gene assays, allele-specific expression (ASE) analysis, TF knockdown, CRISPR/Cas9-mediated genome editing, and expression quantitative trait loci (eQTL) analysis. Finally, we overexpressed *PACS1* (whose expression was most significantly associated with the identified functional SNPs rs10896081 and rs3862386) in mouse primary cortical neurons to investigate if *PACS1* affects dendritic spine density.

**Results:**

We identified 16 functional SNPs (in 9 risk loci); these functional SNPs disrupted the binding of 7 TFs, for example, CTCF and REST binding was frequently disrupted. We then identified the potential target genes whose expression in the human brain was regulated by these functional SNPs through eQTL analysis. Of note, we showed dysregulation of some target genes of the identified TF binding–disrupting SNPs in BD patients compared with controls, and overexpression of *PACS1* reduced the density of dendritic spines, revealing the possible biological mechanisms of these functional SNPs in BD.

**Conclusions:**

Our study identifies functional SNPs in some reported risk loci and sheds light on the regulatory mechanisms of BD risk variants. Further functional characterization and mechanistic studies of these functional SNPs and candidate genes will help to elucidate BD pathogenesis and develop new therapeutic approaches and drugs.

**Supplementary Information:**

The online version contains supplementary material available at 10.1186/s13073-022-01057-3.

## Background

Bipolar disorder (BD) is a severe mental disorder that affects the emotion, cognition, and behaviour of affected individuals, and it affects more than 1% of the world’s population [1, 2]. BD is characterized by recurrent alteration between hypomania and depression, and this disorder is classified into two major clinical subtypes: bipolar disorder type I (BD-I) (which is characterized by hypermanic symptoms) and bipolar disorder type II (BD-II) (which is mainly characterized by hypomanic episodes and severe depression episodes [1]). BD is associated with a high risk of morbidity [3, 4] and mortality (the suicide rate of individuals with BD is approximately 20–30 times higher than that of the general population [5]), which makes it a leading cause of disability worldwide.

Although the pathogenesis of BD remains to be elucidated, converging evidence suggests that both genetic and environmental factors are involved [6, 7]. Environmental risk factors, such as a lack of social support [8], life stress, and sleep-wake cycle disruption, have been reported to have a role in BD [9]. In addition, the high heritability (approximately 80%) indicates the major role of genetic components in BD [10–12]. Over the past decade, several BD risk loci have been identified by genome-wide association studies (GWASs) [13–16]. Despite the great success of these GWASs, to date, the genetic mechanisms of BD (i.e. how risk variants confer risk for BD) remain largely unknown. Considering that most BD risk variants are located in noncoding regions, it is likely that these risk variants confer risk for BD by regulating gene expression [17]. However, pinpointing the functional variants (in the reported risk loci) and elucidating their roles in BD remain major challenges (due to the complexity of linkage disequilibrium (LD) and gene regulation).

To highlight the functional (or causal) risk variants (in the reported BD risk loci) and to elucidate their roles in BD, we performed the first functional genomics study of BD. We first systematically identified risk SNPs that disrupted the binding of TFs (these SNPs were referred to as functional SNPs) by integrating chromatin immunoprecipitation sequencing (ChIP-Seq) and position weight matrix (PWM) data. We then conducted a series of experiments (including reporter gene assays, allele-specific expression (ASE) analysis, TF knockdown, and CRISPR/Cas9-mediated genome editing) to validate the regulatory effects of the identified functional SNPs. We further identified the potential target genes whose expression in the human brain was regulated by the identified functional SNPs using eQTL analysis. Finally, we investigated the function of *PACS1* (a potential target gene regulated by the identified TF binding–disrupting SNPs rs10896081 and rs3862386) and found that overexpression of *PACS1* affected the density of dendritic spines, suggesting the potential mechanism of this gene in BD. Overall, we systematically identified the functional SNPs in the reported risk loci and characterized the regulatory mechanisms of the identified functional SNPs. In addition, our study linked the functional SNPs to their potential target genes, providing a starting point for further functional characterization and development of therapeutic drugs.

## Methods

Information about the reagents and kits used in this study is provided in the Additional file 1.

### Bipolar GWAS used in this study

The genome-wide summary statistics used in this study were obtained from a recent large-scale BD GWAS by Stahl et al. [15]. Stahl et al. first conducted a GWAS on 20,352 BD cases and 31,358 controls (referred to as the discovery stage). Variants with *P* < 1×10^−4^ in the discovery stage were then replicated in an additional cohort (9412 cases and 137,760 controls). A total of 30 genome-wide significant (GWS) loci were finally identified by Stahl et al. [15].

### Extraction of SNPs in LD with the index SNPs

For each risk locus, we extracted SNPs in LD (*r*^2^ > 0.6) with the reported index SNPs using genotype data of Europeans from the 1000 Genomes project [18]. Considering that a wide range of LD values (*r*^2^) were used across the studies to define whether SNPs of interest were in LD with the reported index SNPs [19–23], we conducted an extensive literature search to select a proper LD threshold in this study. Based on our literature search and the following considerations, we selected the widely accepted LD value (*r*^2^ > 0.6) in this study. First, *r*^2^ > 0.6 was widely used to define whether SNPs of interest were in LD with the reported index SNPs [24–37]. Second, we accounted for both the number of included SNPs and the degree of LD. A higher *r*^2^ (e.g. 0.8) reduces the number of included SNPs and leads to the omission of some potential functional SNPs. Finally, in some cases, the functional SNPs might be in low LD with the reported index SNPs [38–40]. We thus selected the widely used *r*^2^ threshold (*r*^2^ > 0.6) in this study. PLINK was used to calculate the LD values and extract the SNPs in LD with the reported index SNPs [41]. In total, 2775 SNPs (including the index SNPs and SNPs in LD with the index SNPs) were extracted (Additional file 2, Table S1).

### Identification of SNPs that affected TF binding

**Fig. 1:**
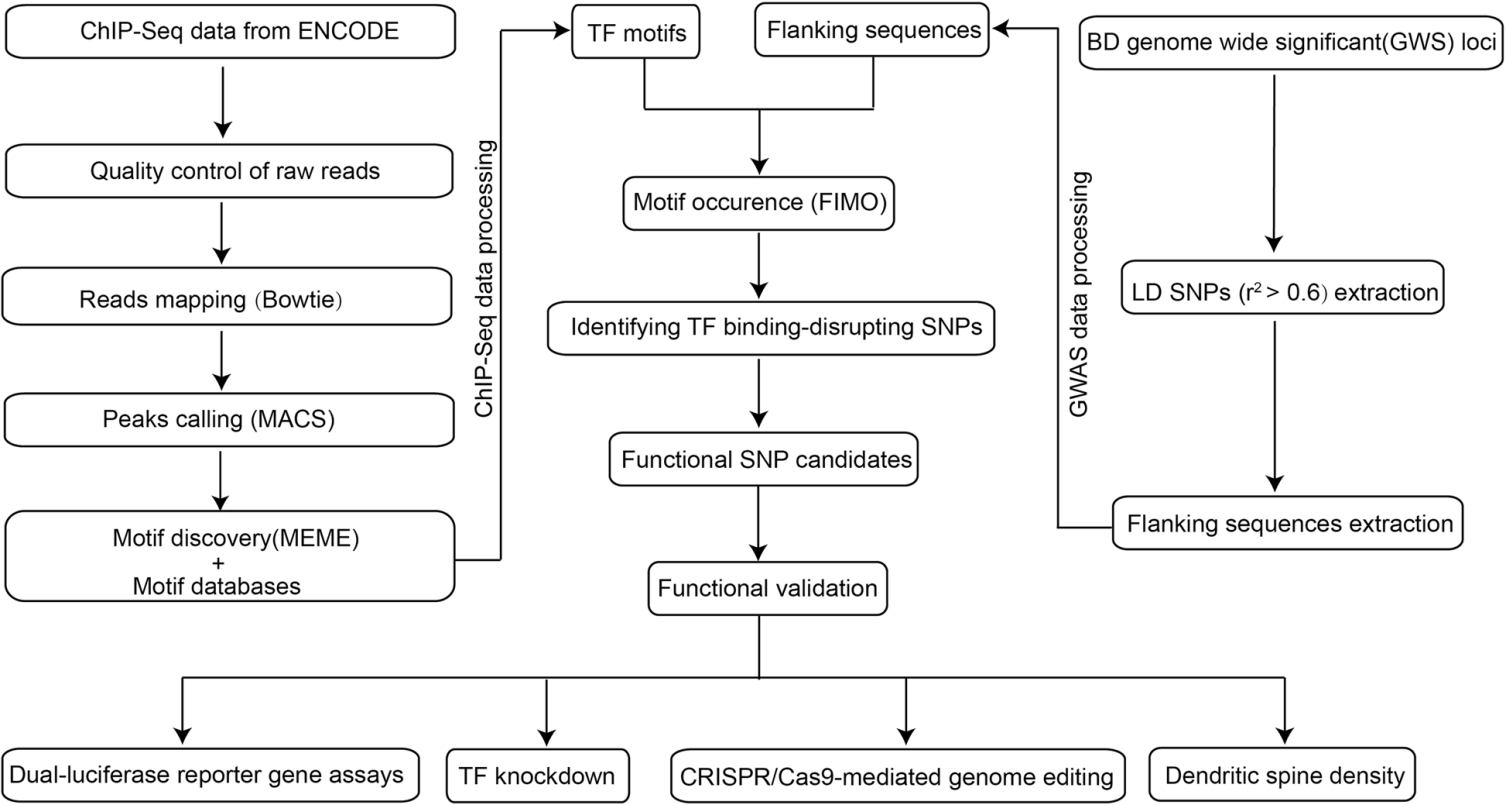
Overview of the study design. Left panel: Data from ChIP-Seq (conducted in neuronal tissues and cell lines) of 34 TFs from the ENCODE project were downloaded. After quality control, read mapping, and peak calling, binding motifs of the included TFs were derived. Right panel: SNPs in LD with the reported index SNPs were extracted (*r*^2^ > 0.6) using genotype data for Europeans from the 1000 Genomes project. FIMO was used to evaluate motif occurrence in the sequences containing the SNPs of interest (sequences (±20 bp) flanking the SNPs of interest were used). A total of 16 functional candidate SNPs were selected, and a series of functional studies was performed to verify their regulatory functions

To identify functional (or potential causal) SNPs in the reported GWS loci, we used a functional genomic approach, which has been described in detail in previous studies [42–44]. The flowchart of our functional genomic-based approach includes three major steps (Fig. 1). The first step is derivation of the TF binding motifs. We downloaded raw data for ChIP-Seq of 34 TFs (conducted in brain tissues or neuronal cell lines from the ENCODE project) (https://www.encodeproject.org/) [45] and conducted a series of analyses. After cleaning by Btrim (http://graphics.med.yale.edu/trim/) [46], the cleaned reads for 30 TFs (4 TFs were excluded because of low quality) were mapped to the reference genome (hg19) by bowtie (http://bowtie-bio.sourceforge.net/index.shtml) [47]. The mapped sam files were then converted into bam files by SAMtools (http://samtools.sourceforge.net) [48]. The derived bam files were used for peak calling (by using MACS (http://liulab.dfci.harvard.edu/MACS/) [49]). The peaks were sorted, and the sequences of the top 500 ChIP-Seq peaks for each TF were used to derive the binding motifs with the MEME online tool kit (https://meme-suite.org/meme/tools/meme) [50]. The derived motifs for each TF were then compared with PWMs (compiled by Whitington et al. https://www.ncbi.nlm.nih.gov/geo/query/acc.cgi?acc=GSE70770) [44], and the matching motifs were used for further analysis. The second step is extraction of the SNPs in LD with the reported index SNPs. We extracted sequences covering ±20 bp around the SNPs of interest (including the index SNPs and SNPs in LD with the index SNPs) (based on the human reference genome (hg19)). For each SNP, two DNA sequences were generated with a difference of only one base at the SNP position (i.e. one sequence contained the reference allele, while the other sequence contained the alternative allele). FIMO (https://meme-suite.org/meme/tools/fimo) [51] was used to search for motif occurrence in the DNA sequences (with the *P* value threshold set at < 0.001) [42–44]. The final step is identification of the TF binding–disrupting SNPs. We then defined a SNP as a TF binding–disrupting SNP by the following criteria: First, the sequences surrounding the SNP of interest contained a TF binding motif (e.g. a CTCF binding motif) (motif occurrence revealed by FIMO). Second, we checked whether the corresponding TF bound to the sequence containing the SNP of interest in the ChIP-Seq data (from ENCODE). If a SNP met both of the above criteria (i.e. motif occurrence and binding to the corresponding TF (ChIP-Seq peak data)), this SNP was defined as a TF binding–disrupting SNP.

### eQTL analysis

We examined the associations between the identified functional SNPs and gene expression in the human brain by using the original reported eQTL results for 5 eQTL datasets [52–56]. The PsychENCODE project includes human brain tissues from 1866 individuals. eQTL results were downloaded from the PsychENCODE website (http://resource.psychencode.org/) [52]. The Common Mind Consortium (CMC) (https://www.ncbi.nlm.nih.gov/geo/query/acc.cgi?acc=GSE30272) [53] eQTL data are based on 209 schizophrenia cases and 206 healthy controls, as well as 52 affective/mood disorder (AFF) cases (tissues from the dorsolateral prefrontal cortex were used for gene expression measurements). The Lieber Institute for Brain Development (LIBD2) (http://eqtl.brainseq.org/phase2/eqtl/) [56] eQTL dataset is based on 286 schizophrenia cases and 265 healthy controls (tissues from hippocampus and the dorsolateral prefrontal cortex were used for expression quantification using RNA sequencing). The xQTL (http://mostafavilab.stat.ubc.ca/xQTLServe/) [54] dataset contains eQTL data for 494 individuals (tissues from the dorsolateral prefrontal cortex were used for gene expression quantification). The Genotype-Tissue Expression (GTEx) project (https://gtexportal.org/home/) [55] contains 49 tissues with eQTL data (sample size (*N*) = 836). Thirteen brain tissues were included in our eQTL analysis. More details about the GTEx project can be found in the original paper and on the GTEx website (https://gtexportal.org/home/) [55]. The eQTL results were extracted directly from the original eQTL studies. If the original eQTL results had been subjected to false discovery rate (FDR) correction, only SNP-gene associations with FDR < 0.05 were retained. If the eQTL results did not contain FDR information, SNP-gene associations were adjusted using the Bonferroni correction.

### Allele-specific expression analysis

Allele-specific expression (ASE) analysis is another way to study the regulatory effect of a variant. The imbalance in expression between two different alleles (maternal and paternal) reflects the potential regulatory effect of a variant. RNA sequencing (RNA-Seq) data can be used to estimate the ASE effect. Suppose that an individual is heterozygous (e.g. with Allele1/Allele2) for a specific SNP. If this SNP is located in a transcribed region, RNA-Seq can determine the number of reads (read counts) containing either Allele1 or Allele2 (quantified as Count_allele1_ and Count_allele2_). The Count_allele1_/Count_allele2_ ratio is compared with (Count_allele1_+Count_allele2_)/2: (Count_allele1_+Count_allele2_)/2 (no ASE) by a binomial test to determine if the number of reads containing Allele1 is significantly different from the number of reads containing Allele2. ASE data in human brain tissues were downloaded from the GTEx project [55] (Version 8). Among the 16 identified functional SNPs, SNPs showing ASE were extracted directly from the GTEx project. More detailed information about ASE analysis in human brain tissues can be found in the original paper and on the GTEx website (https://gtexportal.org/home/) [55].

To test whether the identified TF binding–disrupting SNPs showed significant ASE, we performed a Binomial test (using the binom.test function implemented in R). The total number of variants reported by GTEx V8 is 46,526,292 [55], and 571,220 variants show significant ASE in brain tissues [55, 57]. We tested whether 4 of the 16 functional SNPs showing ASE were statistically significant by running the command binom.test (4, 16, 571220/46526292) implemented in R. 571220/46526292 is the probability if a random SNP showing significant ASE (randomly selected from all the variants of GTEx panel), and Binomial test will determine whether 4 of the 16 function SNP showing significant ASE is statistically significant.

### Cell culture

Three cell lines (HEK293T, SH-SY5Y, and U251) were originally obtained from the Kunming Cell Bank at the Kunming Institute of Zoology, Chinese Academy of Sciences. HEK293T and U251 cells were cultured in high-glucose DMEM (Gibco, Cat. No: C11995500BT) supplemented with 10% FBS (Gibco, Cat. No: 10091148) and 1% penicillin and streptomycin (100 U/mL). SH-SY5Y cells were cultured in high-glucose DMEM (Gibco, Cat. No: C12430500BT) supplemented with 10% FBS (Gibco, Cat. No: 10091148), 10 mM sodium pyruvate solution (Gibco, Cat. No: 11360070), 1% penicillin and streptomycin (100 U/mL), and 1× minimum essential medium nonessential amino acid solution (Gibco, Cat. No: 11140050). All cells were cultured at 37 °C in 5% CO_2_. All cell lines were confirmed to be mycoplasma-free by regular testing by PCR analysis.

### Reporter gene assays

DNA fragments (approximately 500 bp) located in the promoter regions containing different alleles of the candidate functional SNPs were inserted into the pGL4.11[luc2P] vector (which is used to determine promoter activity); alternatively, the enhancer regions containing different alleles of the candidate functional SNPs were inserted into the pGL3-Promoter vector (which is used to determine enhancer activity). The constructs were validated by Sanger sequencing.

We performed reporter gene assays in three cell lines (HEK293T, SH-SY5Y, and U251) as previously described [42, 43]. HEK293T, SH-SY5Y, and U251 cells were plated into 96-well plates at densities of 3.0 × 10^4^ cells/well, 1.0 × 10^5^ cells/well, and 1.0 × 10^4^ cells/well, respectively. After culture for 12 h, the constructed vectors and the internal control vector (pRL-TK, Promega, Cat. No: E2241) were cotransfected into cells using Lipofectamine™ 3000 (Invitrogen, Cat. No: L3000-015). For HEK293T cells, 100 ng of the constructed vectors and 20 ng of the pRL-TK were used. For SH-SY5Y and U251 cells, 150 ng of the constructed vectors and 50 ng of the pRL-TK vector were used. Forty-eight hours post transfection, luciferase activity was measured by a dual-luciferase reporter gene assay system (Promega, Cat. No: E1960) according to the manufacturer’s instructions. Differences were calculated with two-tailed Student’s *t* test, and the significance threshold was set at *P* < 0.05.

### Knockdown of TFs

We used online short hairpin RNA (shRNA) design tools (http://rnaidesigner.thermofisher.com/rnaiexpress/setOption.do?designOption=shrna&pid=-3105315568901923019) [58] to design shRNAs targeting CTCF, PBX3, and TAF1. The sequences of the shRNAs are provided in Additional file 1, Table S2. The annealed oligos were cloned into the pLKO.1 vector, and the constructs were validated by Sanger sequencing. Lentiviral particles were obtained by cotransfecting the constructed vectors (10 μg) with the envelope plasmid pMD2.G (2 μg, Addgene, Cat. No: 12259) and the packaging plasmid psPAX2 (5 μg, Addgene, Cat. No: 12260) into HEK293T cells. Forty-eight hours post transfection, the supernatant containing the packaged lentiviral particles were collected for SH-SY5Y cell infection. The cells were then subjected to puromycin (2 μg/mL, Sigma, Cat. No: 540222) treatment for 1 week to select the cells with stable expression of the shRNAs of interest. The TF knockdown efficiency was determined by real-time quantitative PCR (RT-qPCR).

### Deletion of genomic sequences containing the identified functional SNPs by CRISPR/Cas9 genome editing

To evaluate whether the target genes of interest are regulated by the genomic regions containing the candidate functional SNPs, we used CRISPR/Cas9 technology to delete the genomic regions containing the target SNPs. We designed a pair of sgRNAs (sgRNA1 and sgRNA2, located upstream and downstream of the target SNP, respectively) for each target SNP using the CRISPR sgRNA Design Tool (https://zlab.bio/guidedesign-resources). The plasmids PX459M and EZ-GuideXH were first linearized with the restriction enzyme BbsI, and sgRNA1 and sgRNA2 were inserted into PX459M and EZ-GuideXH, respectively. After validation by Sanger sequencing, the cassette expressing sgRNA2 from EZ-GuideXH was subcloned into a linearized PX459M plasmid that contained sgRNA1 with the restriction enzymes HindIII and XhoI. The ClonExpress II One Step Cloning Kit (Vazyme, Cat. No: C112-01) was employed to generate the final recombinant plasmid expressing both sgRNA1 and sgRNA2 to perform genome editing in HEK293T cells.

### Real-time quantitative PCR (RT-qPCR) analysis

Total RNA (1 μg) was used as templates for reverse transcription by using the PrimeScript™ RT Kit with gDNA Eraser (Takara, Cat. No: RR047A). The generated cDNA was diluted 1:5 for subsequent RT-qPCR analysis, which was carried out using TB Green™ Premix Ex Taq™ II (Tli RNaseH Plus) (Takara, Cat. No: RR820A) in a QuantStudio™ 12K Flex (Applied Biosystems) instrument or a CFX96 Touch™ Real-Time PCR detection system according to the manufacturers’ instructions. *ACTB* was used as the internal control, and the 2^−ΔΔCt^ method [59] was used to calculate relative gene expression. The significance threshold was set at *P* < 0.05, and differences were calculated with two-tailed Student’s *t* test. Primer sequences are provided in Additional file 1, Table S3.

### Dendritic spine density analysis

#### Animals

Wild-type C57BL/6J mice were purchased from Shanghai Model Organisms Center (http://www.modelorg.com), and the animals were maintained in a quiet, uncrowded temperature-controlled house on a 12-h light/dark cycle (lights on at 08:00 and lights off at 20:00) with ad libitum access to lab chow and water. All experiments were approved by the Animal Ethics Committee of the Kunming Institute of Zoology (License number: SMKX-2021-01-001) and conformed to National Advisory Committee for Laboratory Animal Research guidelines.

#### Culture of mouse cortical neurons

The pregnant C57BL/6J mice were anaesthetized and euthanized using a CO_2_ chamber. Brain tissue was harvested from more than 5 mouse embryos (E16.5–17.5), and the prefrontal cortices were isolated in 1× HBSS. The cortical tissues were digested with papain (Worthington, Cat. No: LS003119) and DNase I (Sigma, Cat. No: D4263-1VL) at 37 °C for 18 min. The digested tissues were then dissociated to obtain single-cell suspensions of neurons. Neurons were seeded into 6-well plates (containing coverslips precoated with poly-D-lysine hydrobromide (Sigma, Cat. No: P6407-5MG; 10 μg/mL)) and cultured in 2 mL of culture medium (neurobasal medium (Gibco, Cat. No: 21103049), 2% B27 (Gibco, Cat. No: 17504044), 1× GlutaMAX^TM^-I (Gibco, Cat. No: 35050061), and 2.5% FBS (Biological Industries, Cat. No: 04-001-1ACS)). After 4 h, the medium was changed to culture medium without FBS (neurobasal medium, 2% B27, and 1× GlutaMAX^TM-^I). Cultures were incubated at 37 °C in a humidified, 5% CO_2_ atmosphere for 14 days. Half of the culture medium was refreshed every 7 days.

#### Plasmid transfection and immunofluorescence staining

The recombinant pCDH constructs for *PACS1* (or control vector (pCDH-GFP)) and Venus vector were cotransfected into cultured neurons (cultured for 14 days) using Lipofectamine 3000. After 3 days, the neurons were first fixed with 4% paraformaldehyde and 4% sucrose dissolved in PBS at room temperature and were then treated with 0.1% Triton X-100 and 2% goat serum in PBS. The neurons were stained with anti-mCherry (GeneTex, Cat. No: GTX128508) and anti-GFP (Abcam, Cat. No: ab13970) antibodies overnight at 4 °C and were then incubated with the corresponding secondary antibodies for 1 h at room temperature.

#### Image acquisition and dendritic spines analyses

Analyses of dendritic spine density were carried out as previously described [60–62]. Briefly, images of fixed neurons expressing GFP or both GFP and mCherry were acquired at random using an LSM 880 confocal microscope (Carl Zeiss) by Z-stack image scanning (41 images, 0.25-μm intervals, 1024 × 1024 pixel resolution) with a ×100 objective and 10× digital zoom. The intense expression of GFP encoded by the Venus vector was employed to outline the morphology of neuronal dendritic spines. NeuronStudio [63, 64] was used to analyse secondary or tertiary dendritic spines, including their shape and density. Data obtained from more than 2 dendrites (total length of 60–100 μm per dendrite) of each neuron were averaged as the result for one neuron to reduce variability. Statistical analysis of the total dendritic spine density between the two groups was performed with two-tailed Student’s *t* test. Spine subtype (mushroom, thin, and stubby) densities were analysed using a 2-way ANOVA. All statistical analyses were performed with GraphPad Prism 8, and the significance level was set at 0.05. All assays were performed in at least two independent experiments.

## Results

### Functional genomics identified 16 TF binding–disrupting SNPs in the reported BD risk loci

**Fig. 2:**
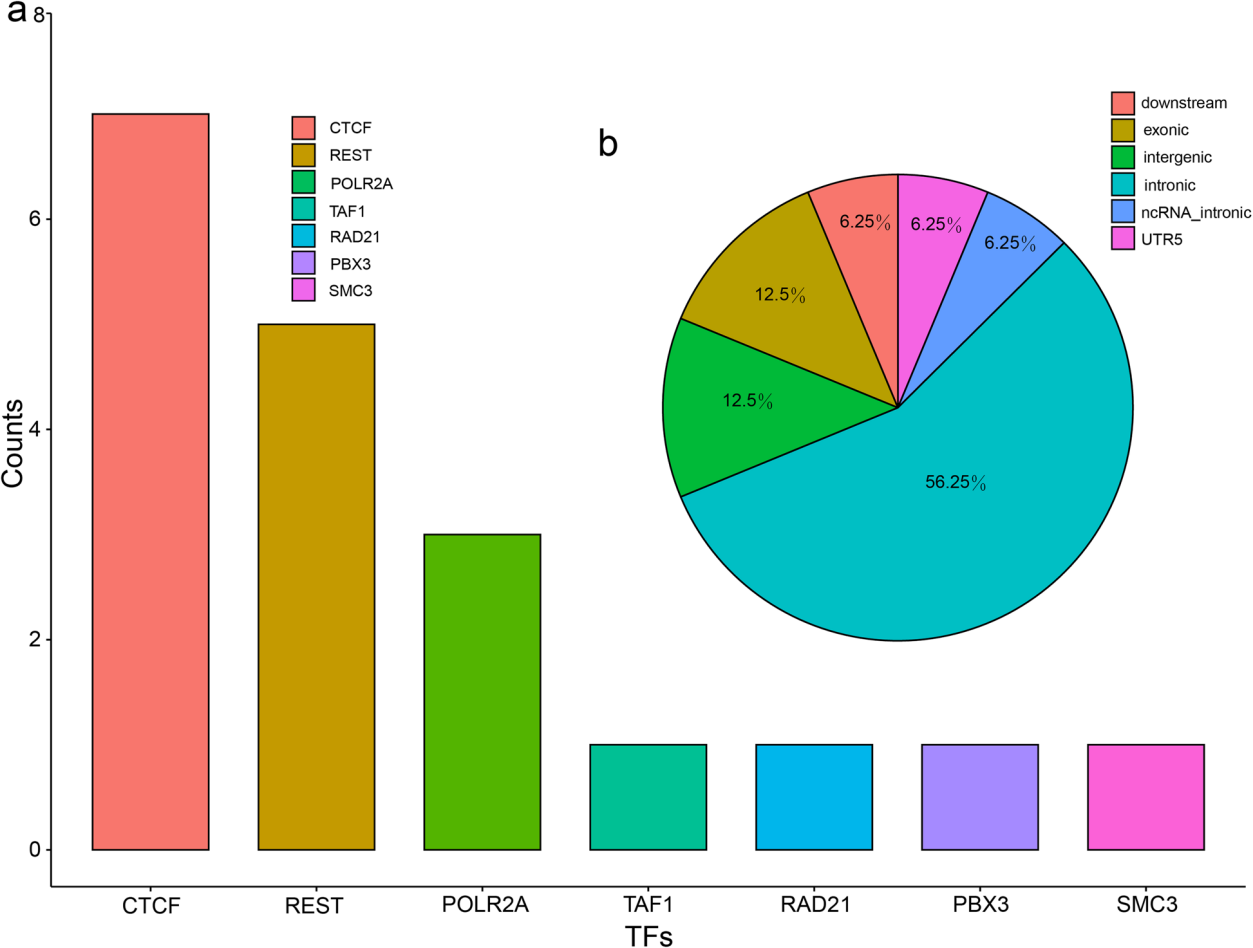
Distribution of the TF binding–disrupting SNPs across the human genome. **a** The number of SNPs that affect the binding affinity of specific TFs. **b** Distribution of the TF binding–disrupting SNPs across the human genome. A large proportion of the functional SNPs disrupt the binding of CTCF, and over half of the functional SNPs are located in intronic regions

To identify the functional SNPs in the reported risk loci, we utilized a functional genomics approach (Fig. 1) [42, 43]. By integrating ChIP-Seq and PWM data, we identified 16 SNPs (Additional file 1, Table S4) that affected the binding of TFs (these TF binding–disrupting SNPs were called functional SNPs). ANNOVAR annotations [65] showed that most of the SNPs were located in intronic (*N* = 9) and intergenic (*N* = 2) regions (Fig. 2). Of note, among the 16 functional SNPs, 7 affected the binding of CTCF, and 5 affected REST binding (Fig. 2). In addition, 3 SNPs affected the binding of two or more TFs: rs2027349 (affected CTCF/TAF1 binding), rs3862386 (affected CTCF/REST binding), and rs228769 (affected CTCF/SMC3 binding) (Additional file 1, Table S4). These results identified functional SNPs in the reported risk loci, suggesting that these functional SNPs may exert their effects on BD by regulating gene expression.

### Reporter gene assays validated the regulatory effects of the identified functional SNPs

**Fig. 3:**
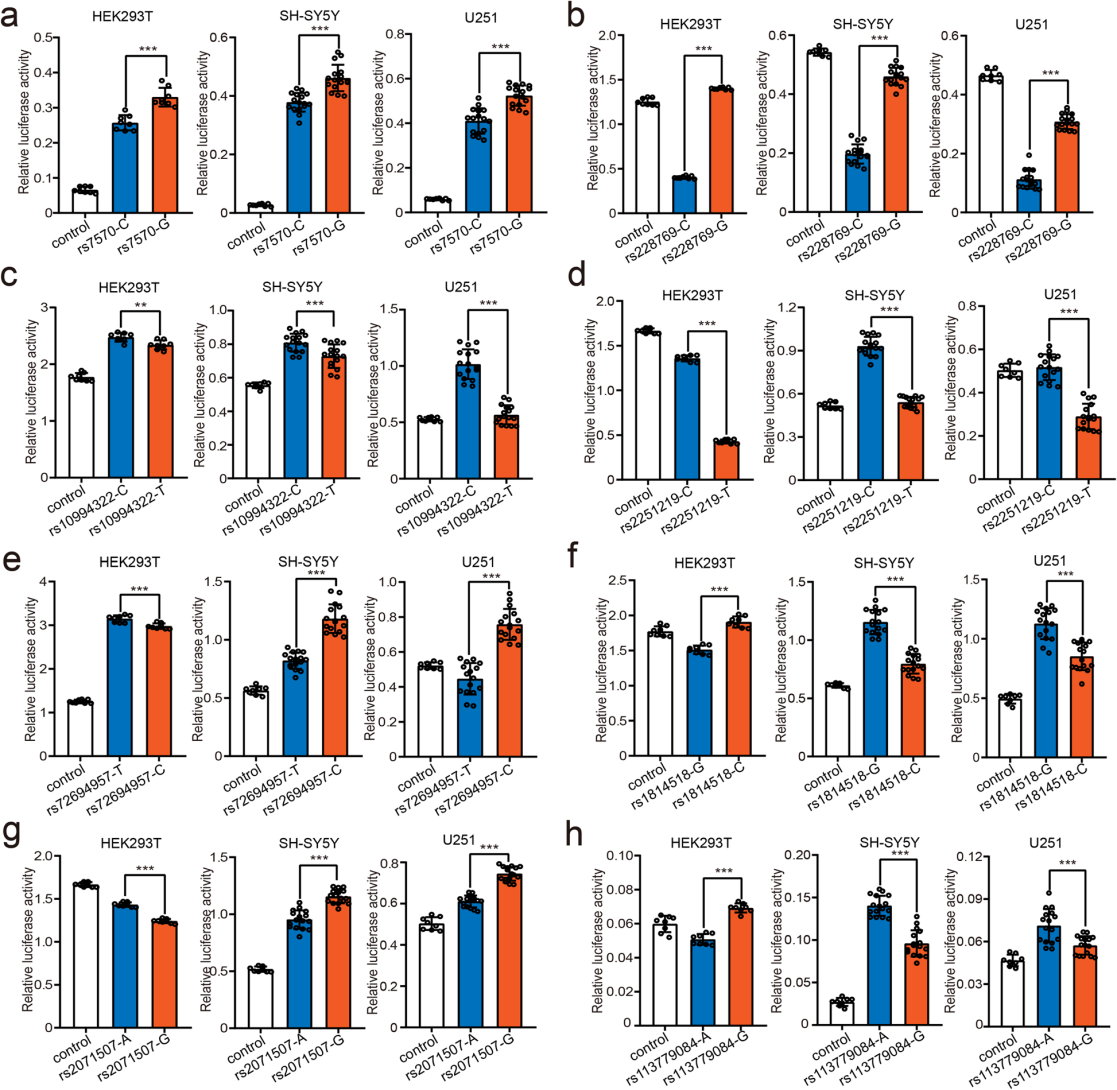
Validation of the regulatory effect of the TF binding–disrupting SNPs. **a** The luciferase expression of the constructs carrying the G allele of rs7570 was significantly higher than that of constructs carrying the C allele in all three tested cell lines. **b** The G allele of rs228769 showed significantly higher luciferase activity than the C allele in all three tested cell lines. **c** The constructs containing the C allele of rs10994322 produced significantly higher luciferase activity than the constructs containing the T allele in all three tested cell lines*.* **d** The constructs carrying the C allele of rs2251219 exhibited significantly higher luciferase activity than the constructs carrying the T allele in all three tested cell lines*.* Notably, this result was inconsistent with the previously published study by Yang et al. [61], likely because of the different lengths (in this study, 513 bp; in Yang et al. study, 435 bp) and directions (in this study, 5′ to 3′; in yang et al. study, 3′ to 5′) of the DNA fragments containing rs2251219 in the pGL3-Promoter vector between the studies. **e** The reporter vectors containing the C allele of rs72694957 showed significantly higher luciferase activity than those containing the T allele in SH-SY5Y and U251 cells. **f** The reporter vectors containing the G allele of rs1814518 displayed significantly higher luciferase activity than those containing the C allele in SH-SY5Y and U251 cells. **g** The luciferase expression of the vectors containing the G allele of rs2071507 was significantly higher than that of the vectors containing the A allele in SH-SY5Y and U251 cells. **h** The constructs carrying the A allele of rs113779084 produced significantly higher luciferase activity than those carrying the G allele in SH-SY5Y and U251 cells. *N* = 8 per group for HEK293T cells, *n* = 8 for the control group, and *n* = 16 per experimental group for SH-SY5Y cells and U251 cells. Two-tailed Student’s t test. *P < 0.05, **P < 0.01, ***P < 0.001

**Fig. 4:**
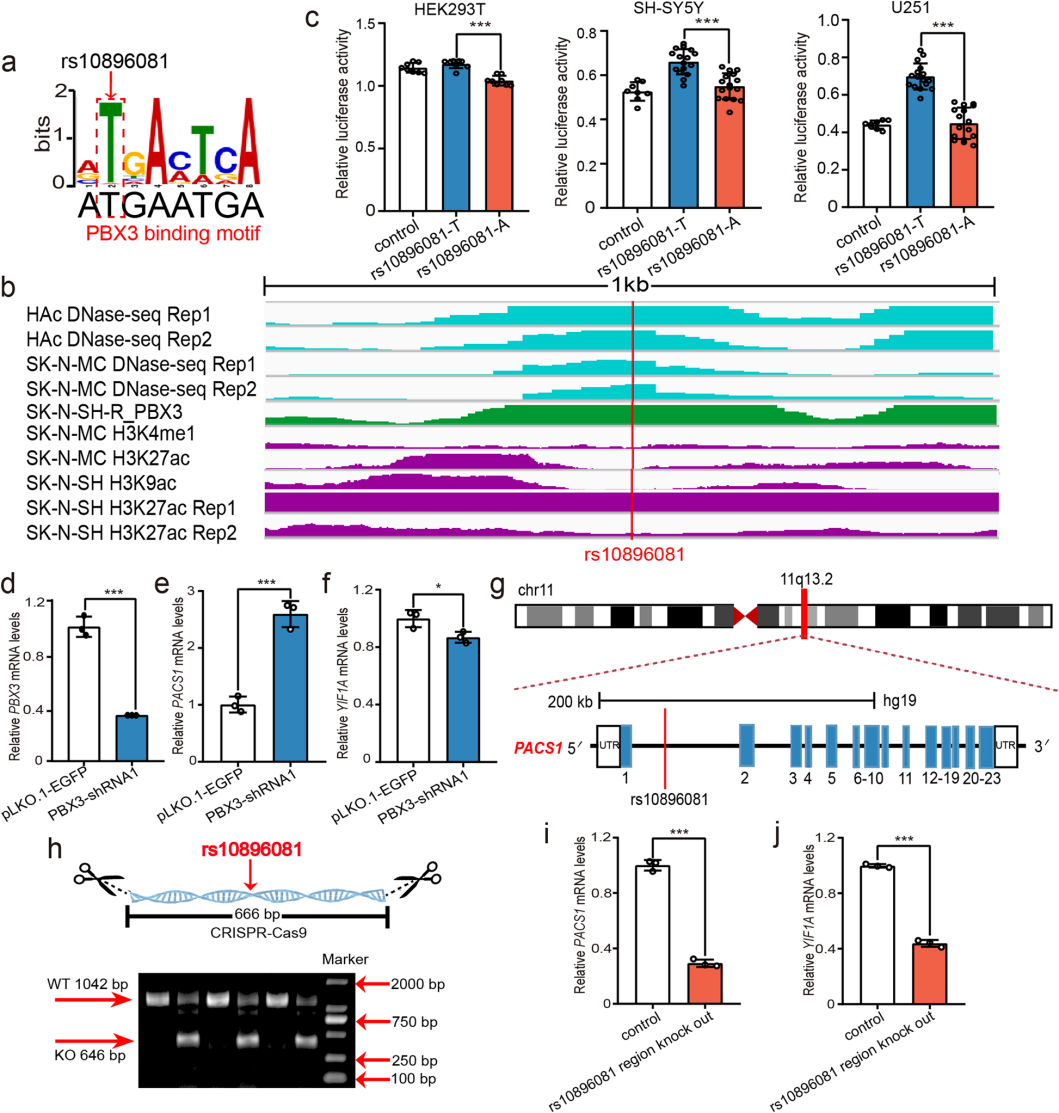
Verification of the regulatory roles of rs10896081. **a** Disruption of PBX3 binding by the SNP rs10896081. **b** ChIP-Seq tracks showing DNase-Seq signals (light blue), TF ChIP-Seq signals (green), and histone modifications (purple) near rs10896081. **c** Reporter gene assays showed that the T allele of rs10896081 produced significantly higher luciferase activity than the A allele in all three tested cell lines. **d–f** Knockdown of PBX3 increased the expression of *PACS1* and decreased the expression of *YIF1A*, indicating that *PACS1* and *YIF1A* are regulated by PBX3. **g** The SNP rs10896081 is located in the first intron of the longest transcript of *PACS1*. **h–j** Deletion of the genomic region containing rs10896081 led to dysregulation of *PACS1* and *YIF1A*. **h** Electrophoretic analysis showed that the segment containing rs10896081 was deleted from the genome. The expected DNA length of rs10896081 in wild-type cells (WT) was 1042 bp, and that in edited cells (KO) was 646 bp. UTR, untranslated region. CDS, coding sequence. *N* = 8 per group for HEK293T cells, *n* = 8 for the control group, *n* = 16 per experimental group for SH-SY5Y and U251 cells, *n* = 3 per group in (**d–f)** and (**h–k)**. Two-tailed Student’s t test. *P < 0.05, **P < 0.01, ***P < 0.001

**Fig. 5:**
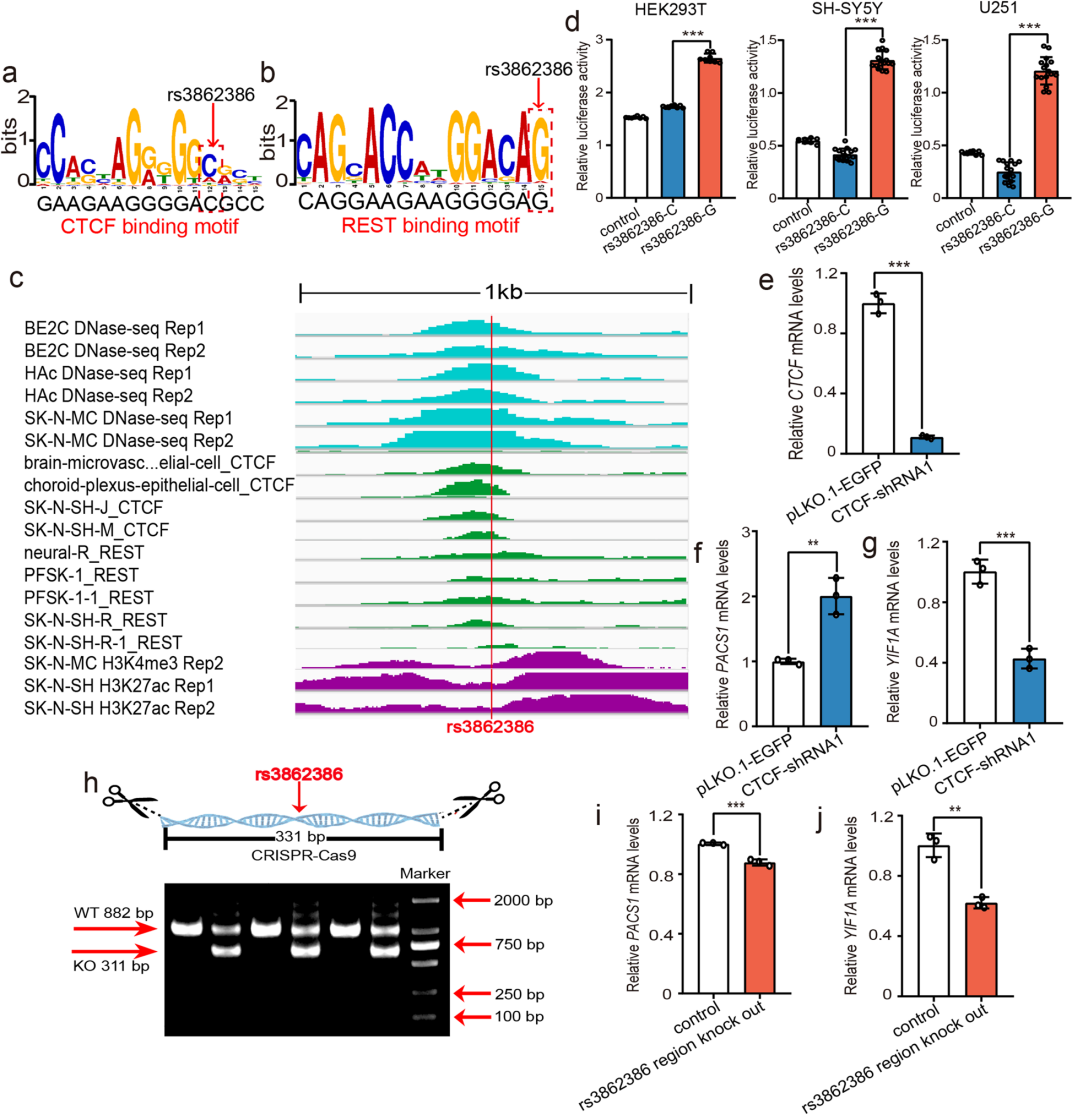
Confirmation of the regulatory effect of rs3862386. **a, b** The SNP rs3862386 disrupts CTCF and REST binding. **c** The SNP rs3862386 is located in a genomic region with DNase-Seq, ChIP-Seq, and histone modification signals, indicating that it is located in a genomic region with active transcription in neuronal cells. **d** Reporter gene assays showed that the G allele of rs3862386 produced significantly higher luciferase activity than the C allele in all three tested cell lines. **e–g** The expression of *PACS1* and *YIF1A* was significantly altered by CTCF knockdown. **h–j** A 331-bp genomic sequence containing rs3862386 was deleted by CRISPR/Cas9-mediated genome editing. Deletion of rs3862386 resulted in altered expression of *PACS1* and *YIF1A*. **h** Genomic PCR/electrophoresis results showed deletion of the genomic region containing rs3862386. WT, genomic DNA containing rs3862386 in wild-type cells (882 bp). KO, genomic DNA containing rs3862386 in edited cells (311 bp). *N* = 8 per group for HEK293T cells, *n* = 8 for the control group, *n* = 16 per experimental group for SH-SY5Y and U251 cells, *n* = 3 per group in (**e–h)** and (**j–l)**. Two-tailed Student’s t test. *P < 0.05, **P < 0.01, ***P < 0.001

**Fig. 6:**
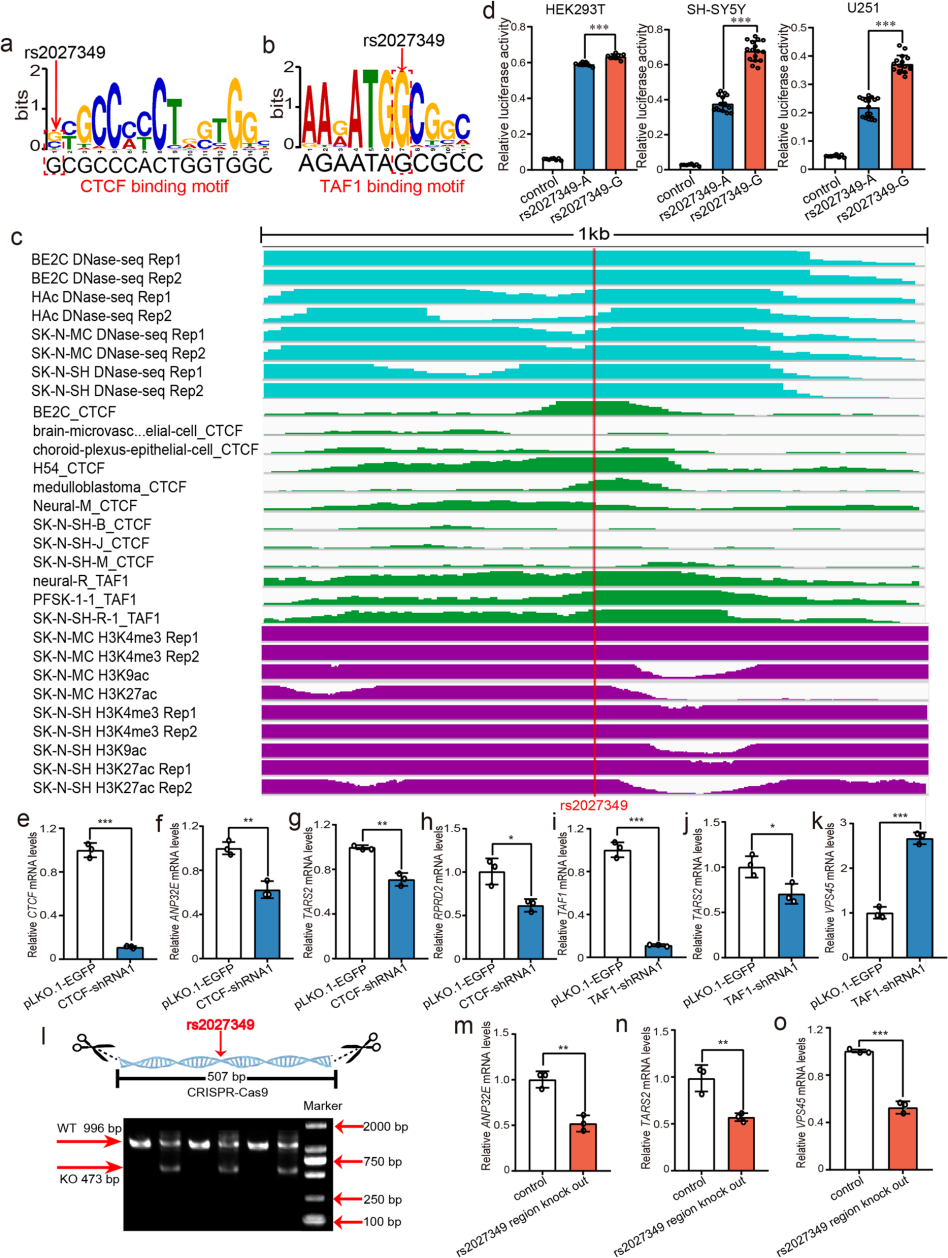
Validation of the regulatory effect of rs2027349 by reporter gene assays, TF knockdown and CRISPR/Cas9-mediated genome editing. **a, b** The SNP rs2027349 disrupts CTCF and TAF1 binding. **c** The 1 kb sequence near the SNP rs2027349 is marked with a strong DNase-Seq (light blue), TF ChIP-Seq (green), and histone modification (purple) signals. **d** Reporter gene assays showed that the G allele of rs2027349 produced significantly higher luciferase activity than the A allele in all three tested cell lines. **e–k** CTCF knockdown led to downregulation of *ANP32E*, *TARS2*, and *RPRD2*. However, knockdown of TAF1 resulted in downregulation of *TARS2* and upregulation of *VPS45.* **l–o** A genomic sequence containing rs2027349 was deleted by CRISPR/Cas9-mediated genome editing, which resulted in downregulation of *ANP32E*, *TARS2*, and *VPS45* expression. **l** Electrophoresis showed that the genomic region containing rs2027349 was deleted by the sgRNAs. WT, genomic DNA containing rs2027349 (996 bp) in wild-type cells. KO, genomic DNA containing rs2027349 (473 bp) in edited cells. *N* = 8 per group for HEK293T cells, *n* = 8 for the control group, *n* = 16 per experimental group for SH-SY5Y and U251 cells, *n* = 3 for each group in (**e–k)** and (**l–o)**. Two-tailed Student’s t test. *P < 0.05, **P < 0.01, ***P < 0.001

Our functional genomic analysis identified 16 TF binding–disrupting SNPs in the reported BD risk loci. To verify the effects of the identified functional SNPs, we carried out dual-luciferase reporter gene assays in HEK293T, SH-SY5Y, and U251 cells. Among the 16 TF binding–disrupting SNPs, all exhibited regulatory effects (i.e. different alleles of these SNPs significantly affected luciferase activity) in at least one tested cell lines (Additional file 1, Table S5). Of note, 11 SNPs exhibited regulatory effects in all three cell lines (Figs. 3, 4, 5, and 6), and 3 SNPs showed regulatory effects in both SH-SY5Y and U251 cells (Additional file 1, Figure S1). These results validated the regulatory effects of these identified functional SNPs.

### Disruption of PBX3 binding by rs10896081

To further investigate the regulatory mechanisms of the functional SNPs, we focused on SNP rs10896081 (located at 11q13.2), which disrupted the binding of the TF PBX3 (Fig. 4a). The ChIP-Seq data indicated that PBX3 can bind to the genomic region containing rs10896081 (Fig. 4b). The DNase-Seq data showed that rs10896081 is located in a genomic region with active transcription in brain tissues (or neuronal cell lines) (Fig. 4b). The histone modification data revealed that rs10896081 is located in a chromosomal region marked with H3K27ac (a marker for active enhancers [66, 67]) (Fig. 4b). These data indicated that rs10896081 is located in an active regulatory element. To test whether rs10896081 has a functional consequence, we performed reporter gene assays. We found that the T allele of rs10896081 produced significantly higher activity than the A allele in all three tested cell lines (Fig. 4c), supporting the regulatory effect of rs10896081. We then conducted eQTL analysis to identify genes whose expression levels in the human brain are associated with rs10896081. Four genes (*PACS1*, *RP11-755F10.1*, *RAB1B*, and *YIF1A*) showed the most significant associations with rs10896081 in the human brain (Additional file 2, Table S6), suggesting that these genes were potential target genes of rs10896081.

The finding that rs10896081 disrupts the binding of PBX3 implies that rs10896081 regulates its eQTL genes by affecting PBX3 binding. To further validate whether *PACS1*, *RP11-755F10.1*, *RAB1B*, and *YIF1A* are regulated by PBX3, we repressed *PBX3* expression in SH-SY5Y cells. PBX3 knockdown resulted in significant dysregulation of *PACS1* and *YIF1A* (Fig. 4d–f), indicating regulatory effects of PBX3 on these two genes. The expression of *RP11-755F10.1* was not examined, as it is a pseudogene. In addition, *RAB1B* did not show a significant expression change in cells with PBX3 repression. As rs10896081 is located in the first intron of the longest transcript of *PACS1* (Fig. 4g), we further investigated the regulatory effect of the genomic region containing rs10896081 on *PACS1* and *YIF1A* using CRISPR/Cas9. Deletion of the genomic sequence (666 bp) containing rs10896081 (Fig. 4h; Additional file 1, Figure S2) led to significant decreases in the expression of *PACS1* and *YIF1A* (Fig. 4i, j). Taken together, these results suggested that rs10896081 regulates the expression of *PACS1* and *YIF1A* by interacting with PBX3.

### Regulatory mechanisms of rs3862386

We also investigated the regulatory mechanism of rs3862386, a SNP that affects the binding of CTCF and REST (Fig. 5a, b). SNP rs3862386 is located in a genomic region with strong ChIP-Seq, DNase-Seq, and histone modification signals (Fig. 5c), indicating that rs3862386 lies in a regulatory element to which the TFs CTCF and REST bind. Reporter gene assays showed that the G allele (protective allele) of rs3862386 was associated with higher luciferase activity than the C allele in all three tested cell lines, confirming the regulatory effect of rs3862386. Interestingly, rs3862386 is in strong LD (*r*^2^ = 0.97) with rs10896081 (a SNP that disrupts the binding of PBX3). To explore the potential target genes regulated by rs3862386, we conducted eQTL analysis and found that rs3862386 was associated with the expression of *PACS1*, *RP11-755F10.1*, *RAB1B*, and *YIF1A* in the human brain (Additional file 2, Table S6). We thus further investigated whether rs3862386 and its binding TFs (CTCF and REST) regulate the expression of the four eQTL genes of rs3862386. Knockdown of CTCF in SH-SY5Y cells resulted in significant upregulation of *PACS1* and downregulation of *YIF1A* (Fig. 5e–g), suggesting that the expression of these genes is regulated by CTCF. However, no alteration in *RAB1B* expression was detected. The expression of *RP11-755F10.1* was not determined because it is a proposed pseudogene. We then further analysed whether rs3862386 regulates the expression of *PACS1* and *YIF1A*. Deletion of the genomic sequence (331 bp) containing rs3862386 (Fig. 5h; Additional file 1, Figure S3) led to dysregulation of *PACS1* and *YIF1A* (Fig. 5i, j), indicating the regulatory effect of rs3862386 on *PACS1* and *YIF1A*. These data suggested that rs3862386 may confer risk for BD by modulating *PACS1* and *YIF1A* expression.

### Disruption of CTCF and TAF1 binding by rs2027349

We characterized rs2027349, a SNP that affects the binding of the TFs CTCF and TAF1 (Fig. 6a, b). The ChIP-Seq data revealed that the TFs CTCF and TAF1 can bind to the genomic sequence containing rs2027349 in cell lines from the human brain, and the DNase-Seq and histone modification data showed that rs2027349 is located in an actively transcribed region (Fig. 6c). Reporter gene assays further validated the regulatory role of rs2027349. The G allele of rs2027349 produced significantly higher luciferase activity than the A allele in all three tested cell lines (Fig. 6d), indicating the regulatory function of rs2027349. eQTL analysis showed that rs2027349 was associated with *ANP32E*, *TARS2*, *RPRD2*, and *VPS45* expression in the human brain (uncorrected *P* < 0.01 in at least one eQTL dataset) (Additional file 2, Table S6), suggesting that rs2027349 regulates these genes.

To further explore whether rs2027349 regulates its eQTL genes via interactions with CTCF and TAF1, we knocked down CTCF and TAF1. CTCF knockdown resulted in significant alterations in *ANP32E*, *TARS2*, and *RPRD2* expression, but *VPS45* expression did not change in cells with CTCF repression (data not shown). In addition, TAF1 knockdown led to dysregulation of *TARS2* and *VPS45* (Fig. 6e–k), indicating the regulatory effect of CTCF and TAF1 on these genes. *ANP32E* and *RPRD2* did not show expression changes in TAF1 knockdown cells (data not shown). Finally, rs2027349 deletion (507 bp) led to dysregulation of *ANP32E*, *TARS2*, and *VPS45* (Fig. 6l–o; Additional file 1, Figure S4), supporting the hypothesis that rs2027349 regulates its eQTL genes by interacting with the TFs CTCF and TAF1.

### eQTL analysis identified the potential target genes regulated by the identified TF binding–disrupting SNPs

To further identify the potential target genes regulated by the identified functional SNPs, we used five human brain eQTL datasets. Among the 16 TF binding–disrupting SNPs, 14 were associated with gene expression (uncorrected *P* < 0.01) in at least one brain eQTL dataset (Additional file 2, Table S6), 12 were significantly correlated with gene expression in at least two brain eQTL datasets (Additional file 2, Table S7), 9 exhibited significant associations with gene expression in at least three brain eQTL datasets (Additional file 2, Table S8), and 6 were significantly associated with gene expression in at least four brain eQTL datasets (Table 1). Notably, 3 SNPs showed significant associations with gene expression in all five brain eQTL datasets (Table 1), suggesting that these SNPs may regulate the expression of their target genes. Considering these results collectively, we prioritized the potential target genes regulated by the identified functional SNPs.

**Table 1:** Association significance between the TF binding-disrupting SNPs and gene expression in the human brain tissues(at least four brain eQTL datasets)

SNP	Gene	CMC^**a**^		LIBD2(DLPFC) ^**a**^		LIBD2(Hippo) ^**a**^		xQTL^**a**^		Brain(GTEx) ^**c**^
		***P*** value	FDR	***P*** value	FDR	***P*** value	FDR	***P*** value	FDR^**b**^	***P*** value
rs2071507	GLT8D1	2.02E-08	2.80E-06	2.83E-06	1.86E-04	NA	NA	1.30E-03	4.69E-02	0.000030( Brain - Nucleus accumbens (basal ganglia))
rs2071507	NEK4	1.25E-12	3.32E-10	4.63E-08	4.39E-06	2.93E-06	2.76E-04	2.68E-04	1.28E-02	0.000005(Brain - Cerebellar Hemisphere);
rs3862386	PACS1	2.30E-12	6.51E-10	9.28E-09	9.95E-07	NA	NA	3.69E-10	5.78E-08	7.0e-7( Brain - Cortex);0.000044( Brain - Frontal Cortex (BA9));
rs2251219	NEK4	5.00E-16	2.01E-13	3.71E-17	1.15E-14	8.40E-12	2.07E-09	1.14E-06	1.00E-04	9.9e-7( Brain - Cerebellar Hemisphere);0.0000062(Brain - Cerebellum)
rs6591201	PACS1	5.69E-06	5.67E-04	7.98E-05	3.56E-03	1.93E-05	1.46E-03	4.51E-09	6.05E-07	0.0000010(Brain - Cortex);0.0000044(Brain - Frontal Cortex (BA9));
rs2270448	PACS1	3.66E-09	6.71E-07	1.54E-06	1.08E-04	NA	NA	5.76E-12	1.14E-09	3.9e-7(Brain - Cortex);0.0000068(Brain - Frontal Cortex (BA9));0.000016(Brain - Hypothalamus)
rs10896081	PACS1	2.21E-12	6.29E-10	9.13E-08	8.21E-06	NA	NA	6.60E-10	9.97E-08	0.0000004(Brain - Cortex);0.000030(Brain - Frontal Cortex (BA9));

^a^Brain tissues from the dorsolateral prefrontal cortex (DLPFC) were used in the CommonMind Consortium (CMC) (N=467), the xQTL map of the human brains (xQTL) (N=494) and the Lieber Institute for Brain Development (LIBD2) brain eQTL (N=551). ^b^The FDR were calculate by R function p.adjust(). ^c^FDR value is not available for GTEx eQTL data, but GTEx provided genome-wide empirical *P*-value threshold for each gene that calculated under FDR=0.05, all the GTEx eQTL *P* values listed here had passed the *P*-value threshold (FDR < 0.05)

### ASE analysis supported the regulatory effects of the identified functional SNPs

To further explore the regulatory effects of the TF binding–disrupting SNPs, we used ASE data from the GTEx project (including only brain tissues). The results showed that 4 of the 16 TF binding–disrupting SNPs exhibited ASE in the human brain. The binomial test indicated that 4 out of the 16 SNPs showed ASE was statistically significant compared with the proportion of SNPs showing ASE in the GTEx project (*P* = 3.67 × 10^−5^, Additional file 2, Table S9), indicating that the TF binding–disrupting SNPs are more likely to show ASE. The four ASE SNPs are rs2027349 (Fig. 6), rs2251219 (Fig. 3d), rs1814518 (Fig. 3f), and rs2270448 (Additional file 1, Figure S1a). These ASE results provided further support for the functionality of the TF binding–disrupting SNPs.

### Differential expression analysis of genes (eQTL genes) whose expression was associated with the identified functional SNPs

To further verify whether the identified functional SNPs may confer risk for BD by regulating the expression of their target genes, we examined the expression levels of target genes (genes whose expression levels were associated with the functional SNPs) in the brains of BD cases and controls using the transcriptome data from PsychENCODE [68]. Expression analysis showed that 30 target genes were differentially expressed (uncorrected *P* < 0.05) in the brains of BD cases compared with controls (Table 2). Of note, the expression of 8 genes (*NISCH*, *ZNF14*, *MTARC2*, *CILP2*, *SNX29P2*, *TMEM110*, *KRBOX1*, and *RP11-867G23.10*) was significantly dysregulated in BD cases compared with controls (FDR < 0.05) (Table 2). These results provided further evidence to support the hypothesis that the identified functional SNPs may contribute to the risk of BD by regulating their target genes.

**Table 2:** Differentially expression analysis of genes (eQTL genes) associated with the identified functional SNPs

Ensembl_gene_id	Gene_name	P.value	FDR	Log_**2**_FC
ENSG00000010322	NISCH	0.000652673	0.025719695	0.053824812
ENSG00000087365	SF3B2	0.025271057	0.191498291	0.034375659
ENSG00000092529	CAPN3	0.025012225	0.190376571	-0.125577335
ENSG00000099785	MARCHF2	0.045287401	0.257423271	0.038582022
ENSG00000105708	ZNF14	0.000256993	0.015221847	0.069962199
ENSG00000117791	MTARC2	0.001085525	0.033910493	0.075874475
ENSG00000160161	CILP2	0.000082	0.007972009	0.220446629
ENSG00000163938	GNL3	0.02844909	0.203075523	0.033097846
ENSG00000166136	NDUFB8	0.047600309	0.263534942	0.037699559
ENSG00000167491	GATAD2A	0.028028595	0.201830947	0.041306987
ENSG00000173599	PC	0.020481736	0.171492954	0.044787537
ENSG00000174791	RIN1	0.006004402	0.086305322	0.067733795
ENSG00000175115	PACS1	0.004206371	0.071508307	0.044807186
ENSG00000180071	ANKRD18A	0.01185869	0.127555154	0.131432268
ENSG00000180979	LRRC57	0.048150452	0.264937063	0.035979853
ENSG00000181638	ZFP41	0.023891485	0.18596537	-0.045816011
ENSG00000187664	HAPLN4	0.035640292	0.227131948	0.098804717
ENSG00000189157	FAM47E	0.012030774	0.128710189	0.062403408
ENSG00000198106	SNX29P2	0.001497159	0.040607774	0.105070975
ENSG00000213533	TMEM110	0.00058669	0.024231067	-0.067704389
ENSG00000215256	DHRS4-AS1	0.014319833	0.141778997	0.095963915
ENSG00000240747	KRBOX1	0.000122545	0.009994372	0.234360192
ENSG00000251867	Y_RNA	0.041863676	0.246888247	0.069471479
ENSG00000254510	RP11-867G23.10	0.000869737	0.029881244	-0.291953622
ENSG00000272414	FAM47E	0.005317918	0.08078778	0.102011622
ENSG00000272573	MUSTN1	0.025553164	0.192617262	-0.266328945
ENSG00000273045	C2ORF15	0.022486423	0.179919816	0.067969908
ENSG00000273170	ANKRD18A	0.017391347	0.156333195	0.161747281
ENSG00000273173	SNURF	0.00818448	0.10344798	0.060262523
ENSG00000273291	KRBOX1	0.01933284	0.165860839	0.157290642

### PACS1 overexpression affected dendritic spine density

To further investigate the potential role of the target genes (those regulated by the identified functional SNPs) in BD, we selected *PACS1* for further functional characterization. The expression of *PACS1* was regulated by the TF binding–disrupting SNPs rs10896081 (Fig. 4) and rs3862386 (Fig. 5). In addition, eQTL analysis indicated that *PACS1* expression was associated with rs10896081 and rs3862386 (Additional file 2, Table S6), suggesting that these two functional SNPs may confer risk for BD by regulating *PACS1* expression. Notably, expression analysis showed a trend of significant upregulation of *PACS1* in BD cases compared with controls [68] (*P* = 4.21 × 10^−3^, FDR = 0.072) (Table 2). These convergent and consistent lines of evidence suggest that the functional SNPs rs10896081 and rs3862386 might confer BD risk by regulating *PACS1* expression.

**Fig. 7:**
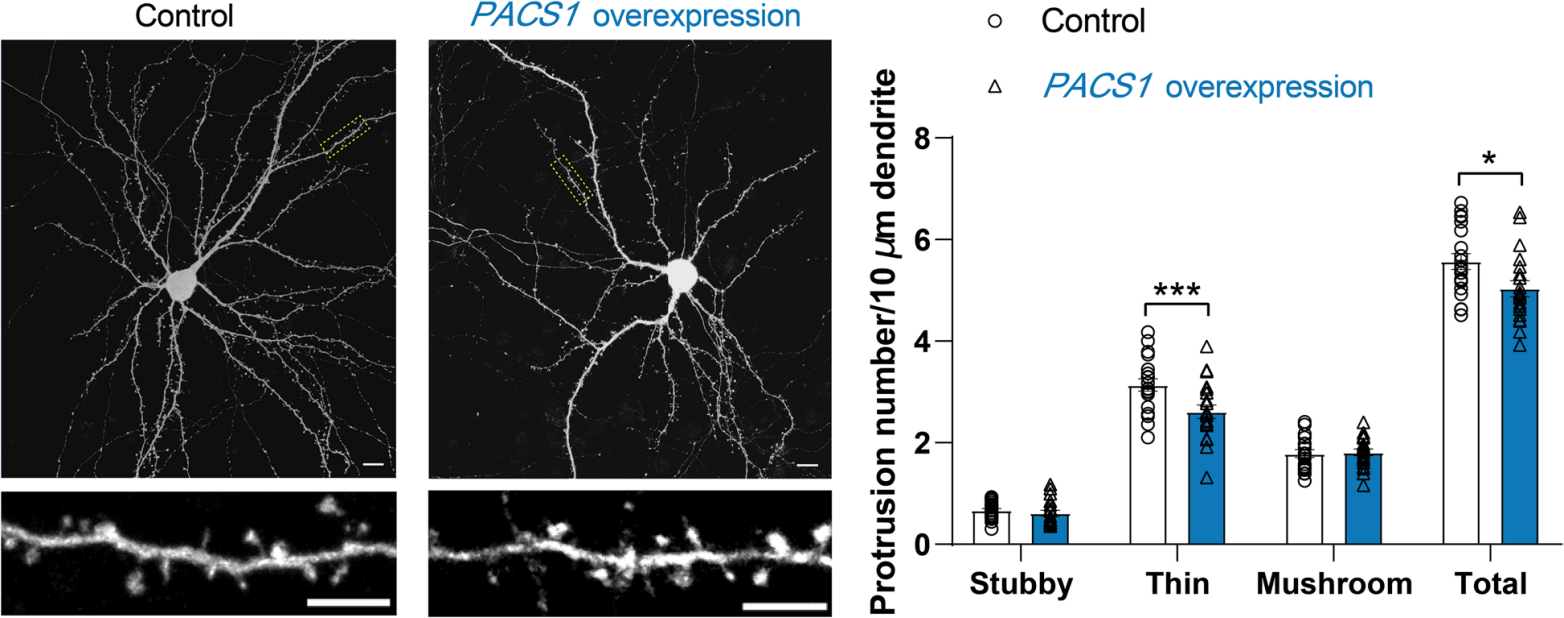
Overexpression of *PACS1* resulted in an altered density of dendritic spines. Representative pictures of cortical neurons transfected with control (pCDH-GFP empty) and *PACS1* overexpression vectors at DIV14. Only the GFP channel is shown to outline dendrite morphology (scale bar 10 μm). Branches of dendrites were imaged in each corresponding neuron (scale bar 5 μm). A two-tailed *t* test was used to determine whether the difference in dendritic spine density was statistically significant. To quantify the density of each dendritic spine subtype, 2-way ANOVA with multiple comparison testing using the Bonferroni correction (*P =* 0.0047) was used. More than 40 dendrites from 20 neurons were analysed in each group (control or *PACS1* overexpression). The error bars indicate the standard error of the mean (SEM) values. *P < 0.05, ***P < 0 .001. Stubby (*P > 0.05*), thin (*P < 0.001*), mushroom (*P > 0.05*)

Accumulating data suggest that dysfunction of dendritic spines (e.g. altered density) may have a pivotal role in BD [17, 69–72]. We thus mimicked the effect of *PACS1* upregulation on dendritic spine density. To gain insights into the function of *PACS1* in in vitro-cultured primary mouse neurons, we cotransfected the plasmid encoding *PACS1* (or the control vector) with the Venus plasmid into mouse cortical neurons (day in vitro (DIV) 14). Notably, we observed a significantly decreased total spine density after overexpression of *PACS1* (control, 5.568 ± 0.691 spines per 10 μm; *PACS1* overexpression, 5.034 ± 0.691 spines per 10 μm; Fig. 7). We further assessed the effects of *PACS1* overexpression on dendritic spines. Neurons transfected with *PACS1* showed a selective decrease in the density of immature thin spines with elongated necks and small heads (control, 3.135 ± 0.536 spines per 10 μm; *PACS1* overexpression, 2.610 ± 0.599 spines per 10 μm; Fig. 7). However, the densities of mushroom and stubby spines were not changed. These results indicate the important role of *PACS1* in mediating the morphogenesis of dendritic spines, suggesting that the identified functional variants rs10896081 and rs3862386 might confer BD risk by modulating *PACS1* expression.

## Discussion

Since the first report of a BD GWAS in 2007 [73], many BD risk loci have been identified through several larger GWASs in the past decade [13, 15, 74–79]. However, due to the complicated LD and the complexity of gene regulation, identifying causal risk variants in the reported risk loci and elucidating the molecular mechanisms of these causal risk variants in the pathophysiology of BD remain major challenges in the post-GWAS era. In this study, we systematically characterized the regulatory mechanisms of BD risk variants using a functional genomic approach. We identified 16 SNPs (from a total of 2775 SNPs) that disrupted the binding of 7 TFs, and we validated the functional consequences of these identified SNPs through a series of assays, including reporter gene assays, ASE analysis, TF knockdown, and CRISPR/Cas9-mediated genome editing. By combining these approaches with eQTL analysis, we further identified potential target genes regulated by these TF binding–disrupting SNPs. Of note, we showed dysregulation of some target genes (regulated by the identified functional SNPs) in BD cases compared with controls. Finally, we investigated the potential role of *PACS1* (regulated by rs10896081 and rs3862386) in BD pathogenesis.

We noted that approximately 43% (7/16) of the TF binding–disrupting SNPs were located in the CTCF binding motif, implicating that altered CTCF binding may be a common mechanism of BD risk variants. Considering the important role of CTCF in regulating chromosomal conformation [80], our data also suggest that a large proportion of BD risk variants may exert their biological effects by regulating the expression of distal genes. In addition, approximately 56% (9/16) of the TF binding–disrupting SNPs were located in intronic regions, demonstrating the important roles of genetic variants in intronic regions in the regulation of BD risk genes.

The ASE results (Additional file 2, Table S9) provide further evidence indicating that our TF binding–disrupting SNPs are regulatory variants. For example, we showed that rs2027349 (Fig. 6) is a regulatory SNP that affects the binding affinity of CTCF and TAF1. ASE analysis also indicated that rs2027349 showed significant ASE in the human brain (data from GTEx V8, Additional file 2, Table S9), further supporting the regulatory role of rs2027349 in neuronal tissues.

We showed that *PACS1* may have a role in BD pathogenesis. Previous studies have shown that dysfunction of dendritic spines might have a role in BD [69]. *PACS1* (phosphofurin acidic cluster sorting protein 1) encodes a trafficking protein that plays a role in the localization of *trans*-Golgi network (TGN) membrane proteins [81]. *PACS1* has been reported to be associated with many diseases, including acquired immune deficiency syndrome (AIDS) [82] and Alzheimer’s disease [83]. It can induce internalization of MHC-I by interacting with the HIV-1 protein Nef, resulting in reduced immune recognition of infected cells [82]. *PACS1* is also involved in the transport of amyloid precursor protein and enhances the formation of brain plaques in Alzheimer’s disease [83]. Mutations in *PACS1* cause a defect in cranial neural crest migration, which leads to intellectual disability [84]. In addition, *PACS1* may play a role in cervical cancer [85]. Although *PACS1* plays an important role in protein transport and is associated with a variety of diseases, the role of this gene in BD is still largely unknown. In this study, we showed that *PACS1* overexpression in mouse primary cortical neurons resulted in an altered density of thin dendritic spines, indicating that *PACS1* may confer risk for BD by affecting the function of dendritic spines.

We noted that some loci contained several TF binding–disrupting SNPs. In our opinion, such TF binding–disrupting SNPs are meaningful for disease susceptibility. First, in our study, we showed that four functional SNPs (rs3862386 (*r*^2^ = 0.99), rs10896081 (*r*^2^ = 0.99), rs2270448 (*r*^2^ = 0.81), and rs6591201 (*r*^2^ = 0.73)) are in LD with the index SNP rs10896090. As shown in Figs. 4 and 5, the results of reporter assays, TF knockdown, and CRISPR/Cas9-mediated genome editing suggested that rs3862386 and rs10896081 might confer risk for BD by modulating *PACS1* and *YIF1A* expression. In addition, we found that different alleles of rs2270448 and rs6591201 resulted in significant differences in luciferase activity in SH-SY5Y and U251 cells (Additional file 1, Figure S1), suggesting that rs3862386 and rs10896081 are functional variants. In fact, many studies have reported that several functional SNPs in a single risk locus might act synergistically or independently to contribute to disease susceptibility. For example, French et al. showed that two functional SNPs (rs78540526 and rs554219) located in enhancer elements conferred risk for breast cancer through regulating the *CCND1* gene [86]. Chatterjee et al. showed that several regulatory variants in enhancer elements conferred risk for Hirschsprung disease by affecting *RET* expression [87]. Shidal et al. [88] showed that the functional variants rs35418111 and rs2078203 (in LD with the index variant in the 21q22.3 risk locus) might be involved in the occurrence of breast cancer by regulating the expression of *YBEY.* In addition to these reports, other studies also have revealed that several functional variants in a specific risk locus contributed to disease susceptibility by modulating the same risk gene [89, 90]. These results suggest that some loci harbour several functional SNPs to regulate the expression of effector risk genes.

There are a few limitations of this study that should be noted. First, only a limited number of TFs (i.e. 30) were included in this study. Considering that the number of TFs in the human genome is approximately 1600 [91], we could not identify the risk SNPs that disrupt the binding of other TFs not included in this study. Second, we only analysed single-nucleotide polymorphisms. However, other types of genetic variations (such as copy number variations (CNVs), chromosomal structural variants, rare mutations, and de novo mutations) may also have a pivotal role in BD. These types of genetic variations were not included in our study, and further work is needed to explain the importance of other types of genetic variations in BD. Third, during execution of this study, a larger GWAS on BD was published [16]. The new risk loci identified in this study were not included in our study. Fourth, overexpression of *PACS1* in cultured mouse primary cortical neurons resulted in a significant decrease in the density of thin dendritic spines, revealing the plausible biological mechanisms of *PACS1* in BD. However, further in vivo analyses (e.g. studies in transgenic mice) are needed to demonstrate the molecular mechanism of *PACS1* in the pathogenesis of BD. Fifth, our findings do not guarantee that the functional SNPs identified in this study are the most relevant SNPs for understanding susceptibility to bipolar disorder. This is a critical limitation of our study. However, considering that pinpointing functional (or causal) variants in the reported risk loci and elucidating their regulatory mechanisms remain challenging in the post-GWAS era, our findings may provide some new insights into the genetic mechanisms of bipolar disorder. Sixth, we characterized only three SNPs (rs10896081, rs3862386, and rs2027349) (Figs. 4, 5, and 6) in detail in our study. The major reasons that we characterized these three SNPs are as follows: (i) The ChIP-Seq data clearly showed that the corresponding TFs bound to genomic sequences containing these three SNPs in human brain tissues or neuronal cells; (ii) these three SNPs were characterized by strong DNase-Seq and histone modification signals; and (iii) the reporter gene assays of these three SNPs showed significant differences between different alleles, with the same effect direction. Characterization of more TF binding–disrupting SNPs will provide more insights into the genetic regulatory mechanisms of BD. Finally, our results suggested that two regulatory SNPs (rs10896081 and rs3862386) might act independently to regulate the potential target gene *PACS1*. However, more work is needed to demonstrate whether these two SNPs act independently or synergistically to regulate *PACS1*.

## Conclusions

In summary, we identified 16 functional SNPs in 9 reported BD risk loci and demonstrated the functional consequences of these SNPs. Our results revealed the complex gene regulatory mechanisms of BD risk variants and provided potential targets for clinical drug development.

## Supplementary Information

**Additional file 1: Supplementary Tables S2-S5 and Supplementary Figures S1-S5.**

**Additional file 2: Supplementary Table S1, Supplementary Tables S6-S9.**

## Data Availability

The PWM data from Whitington et al. are accessible at the NCBI GEO website under the accession number GSE70770 (https://www.ncbi.nlm.nih.gov/geo/query/acc.cgi?acc=GSE70770) [44]. The ChIP-Seq data and histone modification data were downloaded from the ENCODE project (https://www.encodeproject.org/) [45]. The PsychENCODE brain eQTL data were downloaded from the PsychENCODE website (http://resource.psychencode.org/) [52]. The CMC brain eQTL data are accessible at the NCBI GEO website under the accession number GSE30272 (https://www.ncbi.nlm.nih.gov/geo/query/acc.cgi?acc=GSE30272) [53]. The Brain xQTL data were downloaded from the xQTL website (http://mostafavilab.stat.ubc.ca/xQTLServe/) [54]. The GTEx brain eQTL data were downloaded from the GTEx website (https://gtexportal.org/home/) [55]. The LIBD2 brain eQTL data were downloaded from the eQTL website (http://eqtl.brainseq.org/phase2/eqtl/) [56]. The PWM data, ChIP-Seq, DNase-Seq, and histone modification data of the 16 TF binding disrupting SNPs are available in additional file 1, figure S5.

## Abbreviations

GWASs: Genome-wide association studies
BD: Bipolar disorder
ChIP-Seq: Chromatin immunoprecipitation sequencing
SNP: Single-nucleotide polymorphism
ASE: Allele-specific expression
eQTL: Expression quantitative trait loci
TF: Transcription factor
LD: Linkage disequilibrium
PWM: Position weight matrix
GWS: Genome-wide significant
FIMO: Find individual motif occurrences
HEK293T: Human embryonic kidney cell line
SH-SY5Y: Human neuroblastoma cell line
U251: Human glioblastoma cell line
CMC: Common mind consortium
GTEx: Genotype-tissue expression
LIBD: Lieber Institute for Brain Development
xQTL: xQTL map of the human brains
shRNA: Short hairpin RNA
RT-qPCR: Real-time quantitative PCR
KO: Knockout
CNVs: Copy number variations
