# Supplementary material for: Functional genomic analysis delineates regulatory mechanisms of GWAS-identified bipolar disorder risk variants
Source: Genome Med. 2022 May 20;14:53. doi: 10.1186/s13073-022-01057-3 (PMC9121601; doi:10.1186/s13073-022-01057-3)
Supplement: Supplementary file 1 — Additional file 1: Supplementary Tables S2-S5 and Supplementary Figures S1-S5. [file 13073_2022_1057_MOESM1_ESM.docx]

**Supplementary Material for**

**Functional** **genomic analysis delineates regulatory mechanisms of GWAS-identified bipolar disorder risk variants**

Rui Chen^1,3,6^, Zhihui Yang^1,3,6^, Jiewei Liu^1,6^, Xin Cai^1,3,6^, Yongxia Huo^1^, Zhijun Zhang^4,5^, Ming Li^1,*^, Hong Chang^1,*^, Xiong-Jian Luo^1,2,4,*^

**Supplementary information**

**Additional file 1: Supplementary Tables S2-S5 and Supplementary Figures S1-S5.**

**Additional file 2: Supplementary Table S1, Supplementary Tables S6-S9.**

| **KEY RESOURCES TABLE**  **REAGENT or RESOURCE** | **SOURCE** | **IDENTIFIER** |
| --- | --- | --- |
| **Bacterial and virus strains** | | |
| PMD2.G | Addgene | Cat# 12259 |
| PsPAX2 | Addgene | Cat# 12260 |
| **Chemicals, peptides, and recombinant proteins** | | |
| Lipofectamine™ 3000 | ThermoFisher | Cat# L3000015 |
| Dual-luciferase reporter gene assay system | Promega | Cat# E1960 |
| Stbl3 competent cell | Beyotime | Cat# D0378 |
| PEI transfection reagent | Sigma | Cat# 408727 |
| Puromycin | Sigma | Cat# 540222 |
| TRIzol™ LS Reagent | ThermoFisher | Cat# 15596018 |
| GDNA Eraser | TaKaRa | Cat# RR047A |
| TB Green™ Premix Ex Taq™ II | TaKaRa | Cat# RR820A |
| High-glucose DMEM | Gibco | Cat# C11995500BT |
| High-glucose DMEM (no sodium pyruvate) | Gibco | Cat# C12430500BT |
| FBS | Gibco | Cat# 10091148 |
| Sodium pyruvate solution | Gibco | Cat# 11360070 |
| Minimum essential medium nonessential amino acid solution | Gibco | Cat# 11140050 |
| Papain | Worthington | Cat# LS003119 |
| DNase I | Sigma | Cat# D4263-1VL |
| Poly-D-lysine hydrobromide | Sigma | Cat# P6407-5MG |
| Neurobasal | Gibco | Cat# 21103049 |
| B27 | Gibco | Cat# 17504044 |
| GlutaMAXTM-I | Gibco | Cat# 35050061 |
| FBS(Culture of mouse cortical neurons) | Biological Industries | Cat# 04-001-1ACS |
| Anti-mCherry | GeneTex | Cat# GTX128508 |
| Anti-GFP | Abcam | Cat# ab13970 |
| **Critical commercial assays** | | |
| PrimeScript™ RT Kit | TaKaRa | Cat# RR047B |
| Supercompetent Cell Preparation Kit | Beyotime | Cat# D0302 |
| ClonExpress II One Step Cloning Kit | Vazyme | Cat# C112-01 |
| **Deposited data** | | |
| Raw and analyzed data: ChIP-Seq data [1] | Encode | <https://www.encodeproject.org/> |
| Raw and analyzed data: DNase-Seq data | UCSC | <http://hgdownload.cse.ucsc.edu/goldenPath/hg19/encodeDCC/wgEncodeUwDnase/> |
| Raw and analyzed data: Histone modification data [1] | Encode | <https://www.encodeproject.org/> |
| Raw and analyzed data: PWMs data [2] | Whitington et al., 2016.  Accession code: GSE7077 | https://www.ncbi.nlm.nih.gov/geo/query/acc.cgi?acc=GSE70770 |
| PsychENCODE brain eQTL data [3] | PsychENCODE website | <http://resource.psychencode.org/> |
| LIBD2 brain eQTL data [4] | eQTL browser | [http://eqtl.brainseq.org/phase2/eqtl/](http://eqtl.brainseq.org/phase2/eqtl/) |
| Brain xQTL data [5] | xQTL browser | http://mostafavilab.stat.ubc.ca/xQTLServe/ |
| CMC brain eQTL data [6] | Fromer et al., 2016.  Accession code: GSE30272 | https://www.ncbi.nlm.nih.gov/geo/query/acc.cgi?acc=GSE30272 |
| GTEx brain eQTL data [7] | GTEx website | <https://gtexportal.org/home/> |
| **Experimental models: cell lines** | | |
| HEK293T cell | Kunming Cell Bank | N/A |
| SH-SY5Y cell | Kunming Cell Bank | N/A |
| U251 cell | Kunming Cell Bank | N/A |
| **Oligonucleotides** | | |
| ShRNAs sequence; See Table S2 | This paper | N/A |
| RT-qPCR primers; See Table S3 | This paper | N/A |
| **Recombinant DNA** | | |
| PGL4.11[luc2P] | Promega | Cat# E6661 |
| PGL3 promoter | Promega | Cat# E1761 |
| PRL-TK | Promega | Cat# E2241 |
| **Software and algorithms** | | |
| Btrim [8] | Yong Kong, 2011 | [http://graphics.med.yale.edu/trim/](http://graphics.med.yale.edu/trim/) |
| SAMtools [9] | Li et al., 2009 | [http://samtools.sourceforge.net](http://samtools.sourceforge.net/) |
| Bowtie [10] | version 1.1.2 | <http://bowtie-bio.sourceforge.net/index.shtml> |
| MACS [11] | version 1.4 | [http://liulab.dfci.harvard.edu/MACS/](http://liulab.dfci.harvard.edu/MACS/) |
| MEME [12] | Version 5.3.3 | <https://meme-suite.org/meme/tools/meme> |
| FIMO [13] | Version 5.3.3 | https://meme-suite.org/meme/tools/fimo |
| PLINK [14] | version 1.9 | <http://zzz.bwh.harvard.edu/plink/> |

| **Table S2. shRNAs used to knockdown of TFs** | |  |
| --- | --- | --- |
| **Primer name** | **Primer sequence(5'>3')** | |
| Human-*CTCF*-  shRNA-F | CCGGGAAAGATGCGCTCTAAGAAAGCTCGAGCTTTCTTAGAGCGCATCTTTCTTTTTG | |
| Human-*CTCF*-  shRNA-R | AATTCAAAAAGAAAGATGCGCTCTAAGAAAGCTCGAGCTTTCTTAGAGCGCATCTTTC | |
| Human-*PBX3*-  shRNA-F | CCGGGGTTCTTCAGATAACTCTATTCTCGAGAATAGAGTTATCTGAAGAACCTTTTTG | |
| Human-*PBX3*-  shRNA-R | AATTCAAAAAGGTTCTTCAGATAACTCTATTCTCGAGAATAGAGTTATCTGAAGAACC | |
| Human-*TAF1*-  shRNA-F | CCGGGCAAAGATGGTGATCTTATTCCTCGAGGAATAAGATCACCATCTTTGCTTTTTG | |
| Human-*TAF1*-  shRNA-R | AATTCAAAAAGCAAAGATGGTGATCTTATTCCTCGAGGAATAAGATCACCATCTTTGCT | |
| **Note:** CCGG: Age I restriction site; AATT: EcoR I restriction site; CTCGAG: loop; TTTTTG: T-terminate. | | |

| **Table S3. RT-qPCR primers used in this study** | | |
| --- | --- | --- |
| **Primer name** | **Primer sequence(5'>3')** |  |
| Human-*CTCF*-qPCR-F | TTGTCATGCTCGGTTTACCCA |  |
| Human-*CTCF*-qPCR-R | CAATATAGGAATGCTGCTTTCGC |  |
| Human-*TAF1*-qPCR-F | CCACCAAGCTGTTGCCAAGT |  |
| Human-*TAF1*-qPCR-R | CGCCAAACAGATGGGACATT |  |
| Human-*PBX3*-qPCR-F | AAGCAGGACATCGGCGACAT |  |
| Human-*PBX3*-qPCR-R | GCTGAAGAGCGCTGGTTTCAT |  |
| Human-*VPS45*-qPCR-F | GCAAGAGTGACGTGAAGTCAT |  |
| Human-*VPS45*-qPCR-R | GAGCTGATAACGAATCATGGGAC |  |
| Human-*TARS2*-qPCR-F | CCGGCTCCAAGGTTTACAGG |  |
| Human-*TARS2*-qPCR-R | TGTGCCATGCTTGCTAATCTC |  |
| Human-*RPRD2*-qPCR-F | AGTCCTCGTTGGATCGAAAATTC |  |
| Human-*RPRD2*-qPCR-R | CAAACGGTGGGGATATGCAGA |  |
| Human-*ANP32E*-qPCR-F | TGGAAGTCCTGGCAGAGAAATGT |  |
| Human-*ANP32E*-qPCR-R | TCTTCCAGGTTTGTGATCTCACAGT |  |
| Human-*PACS1*-qPCR-F | GTTTAAAGTTTCAGATGAGGTGGGC |  |
| Human-*PACS1*-qPCR-R | TGCTTTCTGTCTCCTCCATCTCAG |  |
| Human-*CTSF*-qPCR-F | CCCTCCAATGCCTACTCGG |  |
| Human-*CTSF*-qPCR-R | CCAGCTTCTGCTCGTTCTG |  |
| Human-*YIF1A*-qPCR-F | CAAGCGGTGGTTATTCCAGC |  |
| Human-*YIF1A*-qPCR-R | CCCCAAGCAAGTGGTTGACA |  |
| Human-*NISCH*-qPCR-F | GAAGGCACAACCCTAGAAGGC |  |
| Human-*NISCH*-qPCR-R | CACAGACTCGTCGATCTCGGA |  |

| **Table S4. 16 TF binding-disrupting SNPs used in this study** | | | | | | |  |
| --- | --- | --- | --- | --- | --- | --- | --- |
| **SNP ID** | | **Chr** | **Ref^a^** | **Alt^b^** | **Location** | **Nearby Genes^c^** | **Disrupted TFs** |
| rs2027349 | | [Chr 1](http://asia.ensembl.org/Homo_sapiens/Location/View?contigviewbottom=variation_feature_variation%3Dnormal%2Cseq%3Dnormal;db=core;r=1:150067571-150067671;source=dbSNP;v=rs2027349;vdb=variation;vf=1317000) | G | A | 150039678 | VPS45 | CTCF/TAF1 |
| rs72694957 | | Chr 1 | C | T | 150135291 | PLEKHO1 | REST |
| rs10994322 | | [Chr 10](http://asia.ensembl.org/Homo_sapiens/Location/View?contigviewbottom=variation_feature_variation%3Dnormal%2Cseq%3Dnormal;db=core;r=10:60376471-60376571;source=dbSNP;v=rs10994322;vdb=variation;vf=26867336) | C | T | 62136279 | ARL4AP1 | RAD21 |
| rs6591201 | | Chr 11 | C | G | 65805970 | CATSPER1 | CTCF |
| rs2270448 | | Chr 11 | G | T | 65835931 | SF3B2 | POLR2A |
| rs10896081 | | [Chr 11](http://asia.ensembl.org/Homo_sapiens/Location/View?contigviewbottom=variation_feature_variation%3Dnormal%2Cseq%3Dnormal;db=core;r=11:66112833-66112933;source=dbSNP;v=rs10896081;vdb=variation;vf=85995255) | A | T | 65880354 | PACS1 | PBX3 |
| rs3862386 | | Chr 11 | G | C | 65894424 | PACS1 | CTCF/REST |
| rs7570 | | Chr 11 | G | C | 66610645 | RCE1 | CTCF |
| rs1814518 | | [Chr 15](http://asia.ensembl.org/Homo_sapiens/Location/View?contigviewbottom=variation_feature_variation%3Dnormal%2Cseq%3Dnormal;db=core;r=15:42581424-42581524;source=dbSNP;v=rs1814518;vdb=variation;vf=16094306) | G | A | 42873672 | STARD9 | CTCF |
| rs228769 | | Chr 17 | C | G | 42193185 | HDAC5 | CTCF/SMC3 |
| rs6503488 | | [Chr 17](http://asia.ensembl.org/Homo_sapiens/Location/View?contigviewbottom=variation_feature_variation%3Dnormal%2Cseq%3Dnormal;db=core;r=17:44168740-44168840;source=dbSNP;v=rs6503488;vdb=variation;vf=87987652) | G | T | 42246158 | HROB | REST |
| rs2965186 | | Chr 19 | G | T | 19497195 | GATAD2A | POLR2A |
| rs75582668 | | [Chr 19](http://asia.ensembl.org/Homo_sapiens/Location/View?contigviewbottom=variation_feature_variation%3Dnormal%2Cseq%3Dnormal;db=core;r=19:19458164-19458264;source=dbSNP;v=rs75582668;vdb=variation;vf=48559886) | C | T | 19569023 | GATAD2A | REST |
| rs2251219 | | Chr 3 | T | C | 52584787 | SMIM4 | POLR2A |
| rs2071507 | | [Chr 3](http://asia.ensembl.org/Homo_sapiens/Location/View?contigviewbottom=variation_feature_variation%3Dnormal%2Cseq%3Dnormal;db=core;r=3:52792641-52792741;source=dbSNP;v=rs2071507;vdb=variation;vf=962192) | G | A | 52826707 | ITIH1 | CTCF |
| rs113779084 | | [Chr 7](http://asia.ensembl.org/Homo_sapiens/Location/View?contigviewbottom=variation_feature_variation%3Dnormal%2Cseq%3Dnormal;db=core;r=7:11832111-11832211;source=dbSNP;v=rs113779084;vdb=variation;vf=28652199) | G | A | 11871787 | THRAP3P3 | REST |
| **Note:** ^a^Reference allele, ^b^Alternative allele. ^c^Nearby genes of the TF binding-disrupting SNPs. | | | | | | | |

| **Table S5. Summary of the reporter gene assays** | | | |
| --- | --- | --- | --- |
|  | If allelic differences at the identified regulatory SNPs affect luciferase activity significantly | | |
| Regulatory SNP id | HEK 293T | SH-SY5Y | SK-N-SH |
| rs2027349 | Yes | Yes | Yes |
| rs72694957 | Yes | Yes | Yes |
| rs10994322 | Yes | Yes | Yes |
| rs6591201 | No | Yes | Yes |
| rs2270448 | No | Yes | Yes |
| rs10896081 | Yes | Yes | Yes |
| rs3862386 | Yes | Yes | Yes |
| rs7570 | Yes | Yes | Yes |
| rs1814518 | Yes | Yes | Yes |
| rs228769 | Yes | Yes | Yes |
| rs6503488 | No | Yes | Yes |
| rs2965186 | Yes | No | No |
| rs75582668 | Yes | No | No |
| rs2251219 | Yes | Yes | Yes |
| rs2071507 | Yes | Yes | Yes |
| rs113779084 | Yes | Yes | Yes |

**
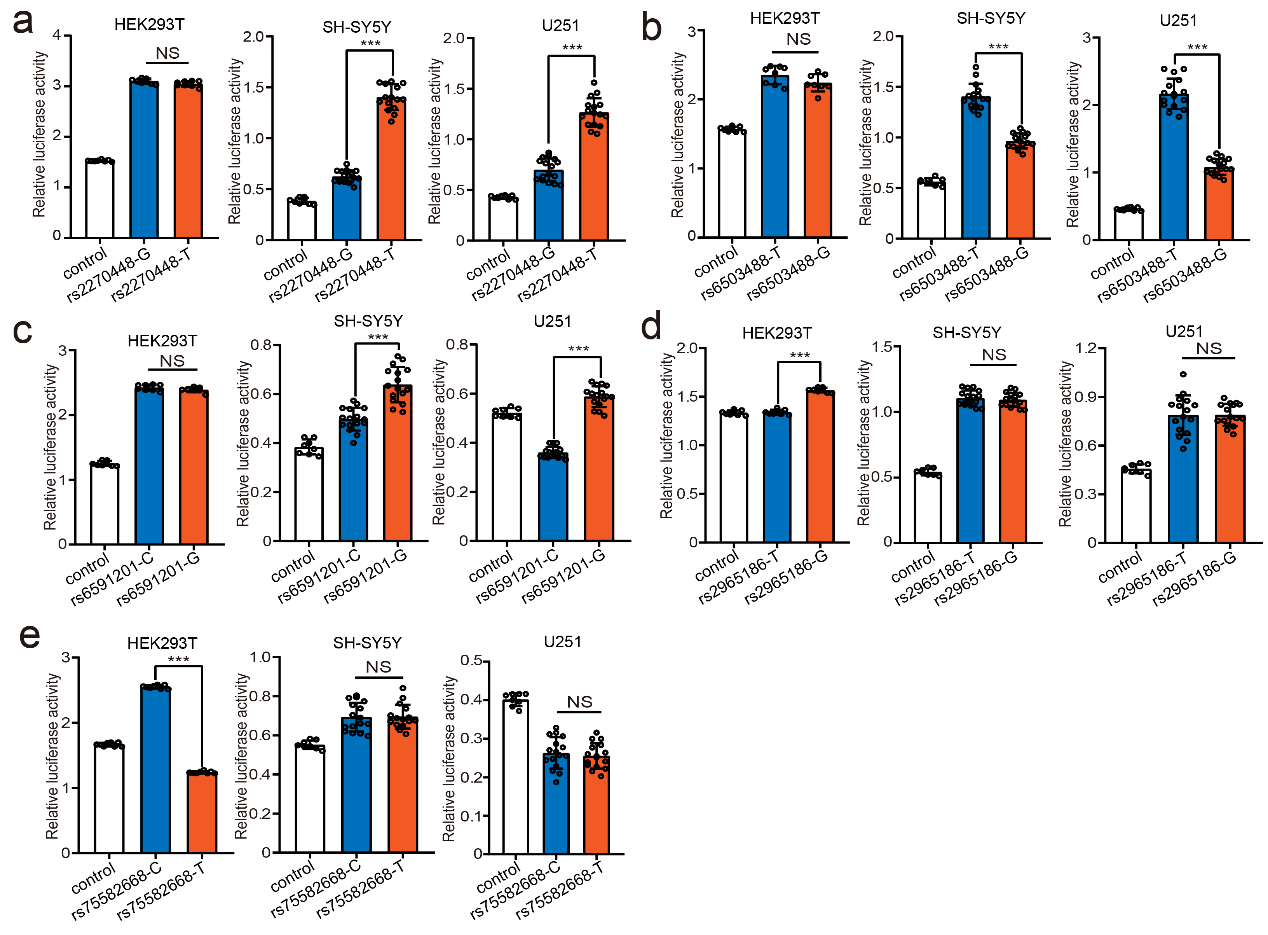
**

**Figure S1. Reporter gene assays validated the regulatory effect of the identified TF binding-disrupting SNPs.** N = 8 for each group in HEK293T cells, and n = 8 for the control group, n = 16 per experimental group for SH-SY5Y and U251 cells. Two-tailed *Student’s t* test was used for statistical analyses. *P < 0.05, **P < 0.01, ***P < 0.001.

**The uncropped gel/full length original blot**

**
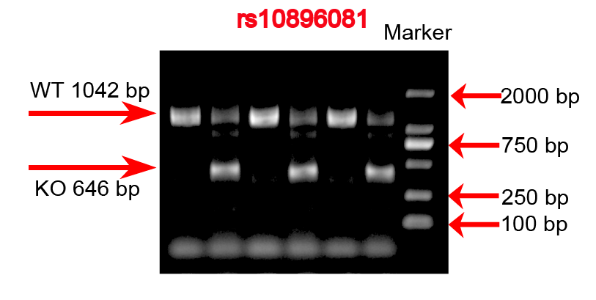
**

Figure S2. Full-length gel blots corresponding to cropped images in Figure 4h within the main text.

**The uncropped gel/full length original blot**

**
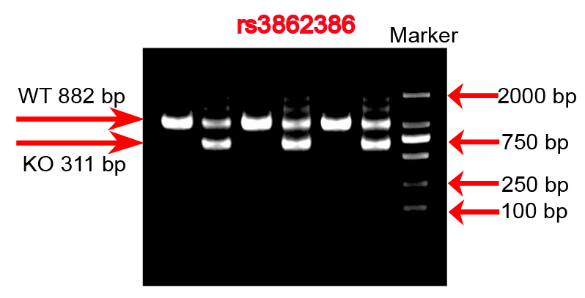
**

Figure S3. Full-length gel blots corresponding to cropped images in Figure 5h within the main text.

**The uncropped gel/full length original blot**


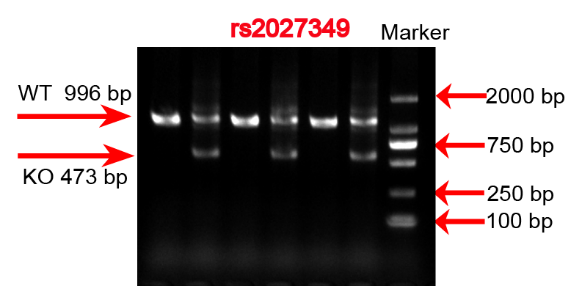


Figure S4. Full-length gel blots corresponding to cropped images in Figure 6l within the main text.


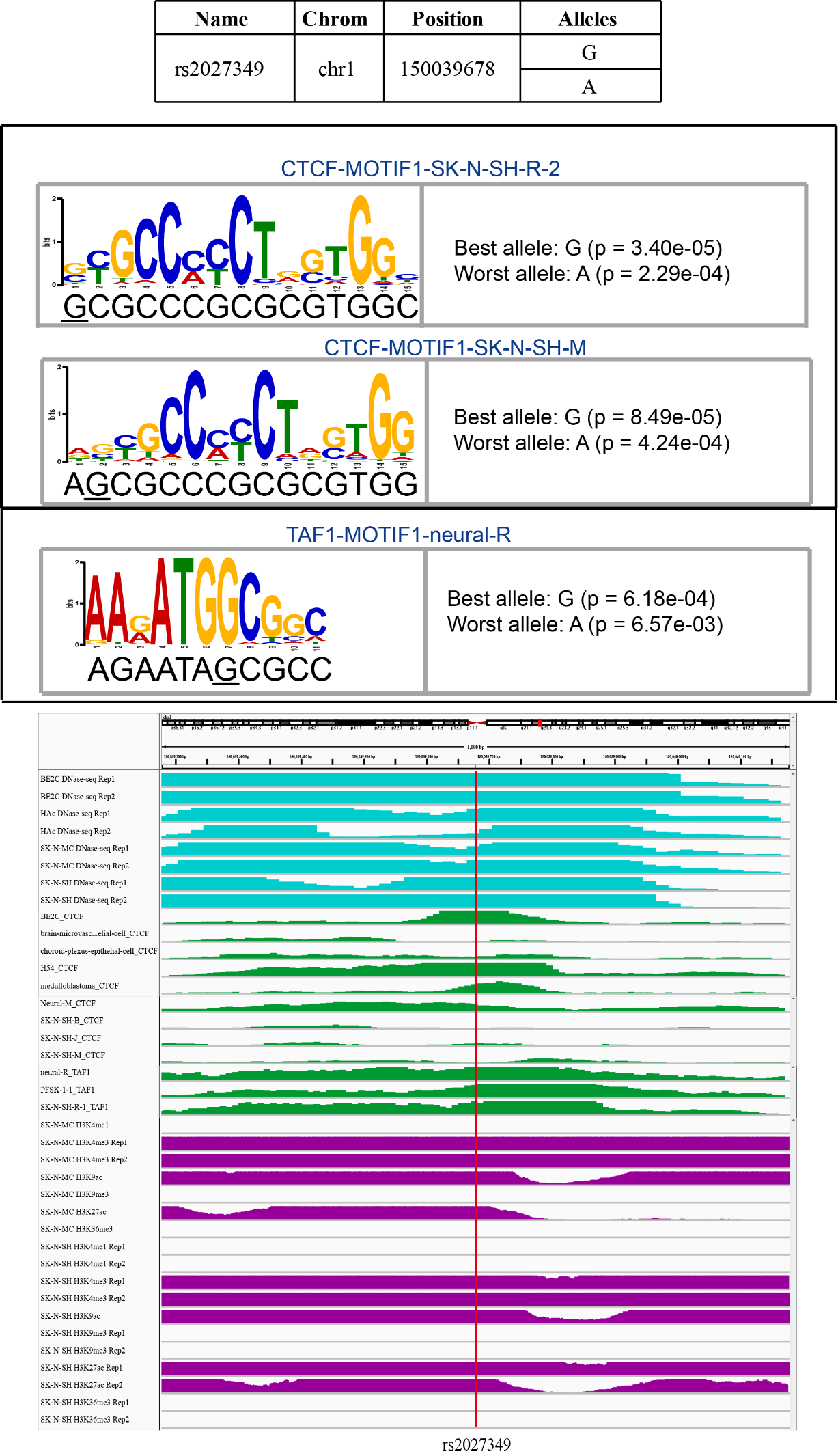


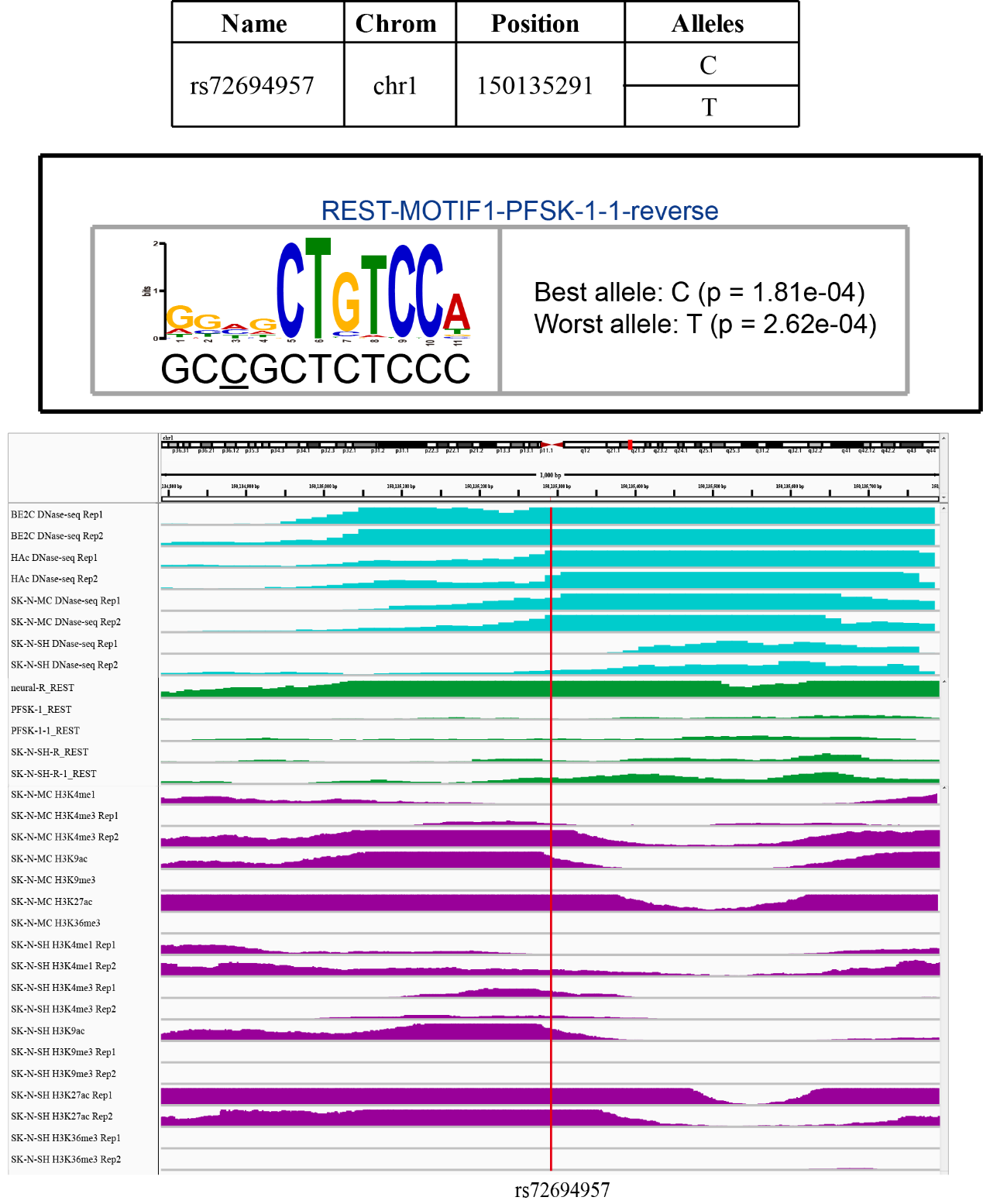


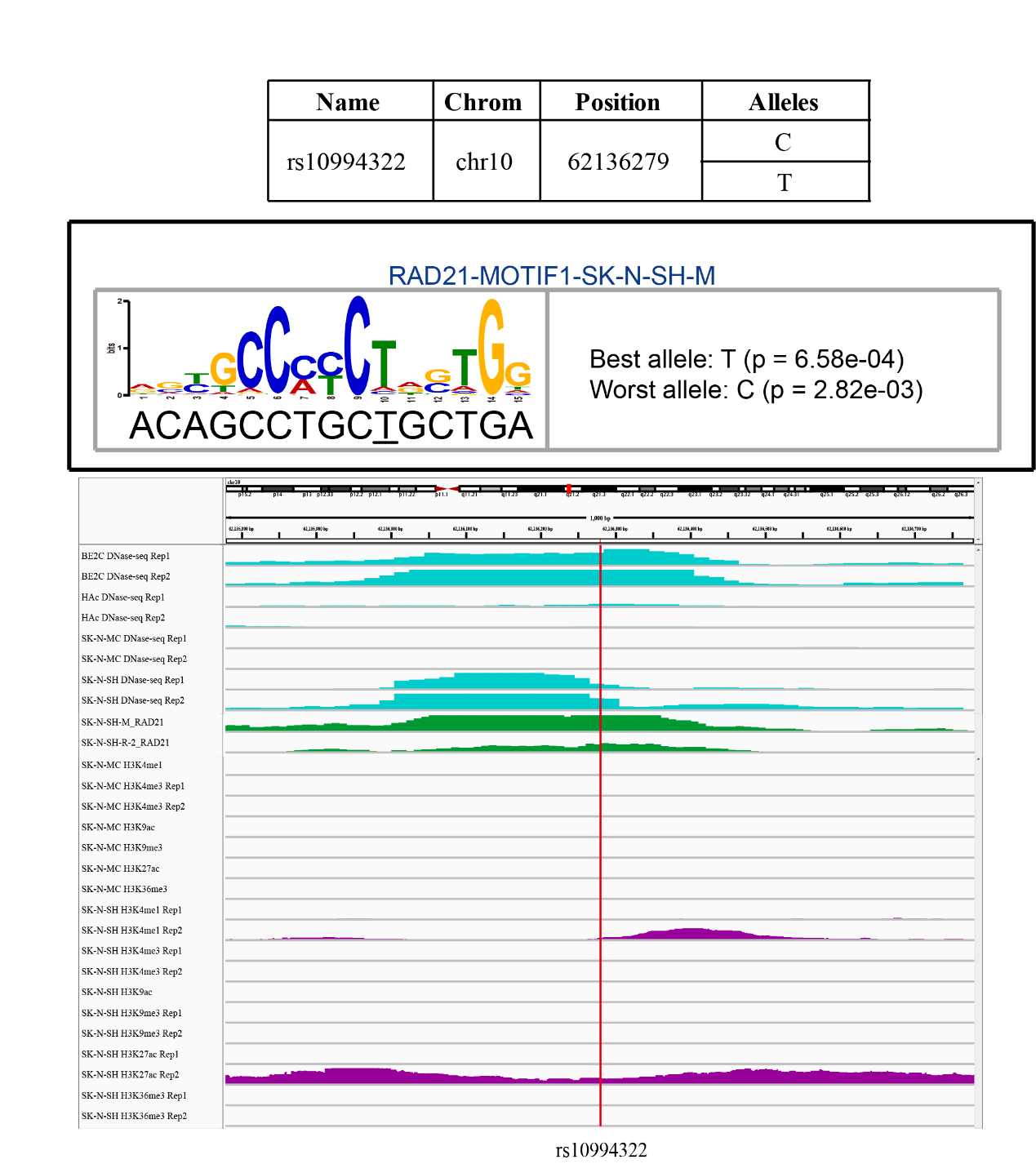


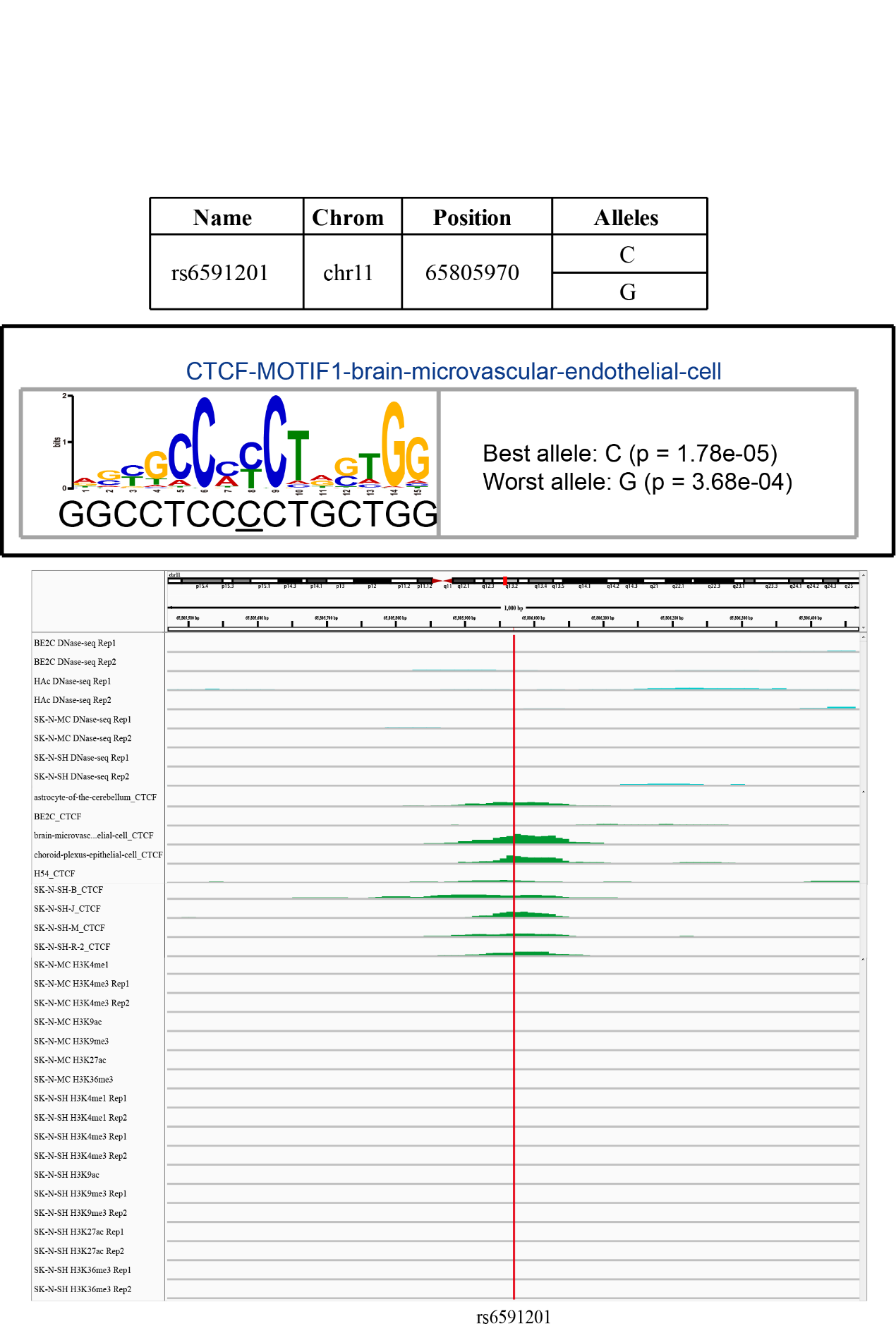

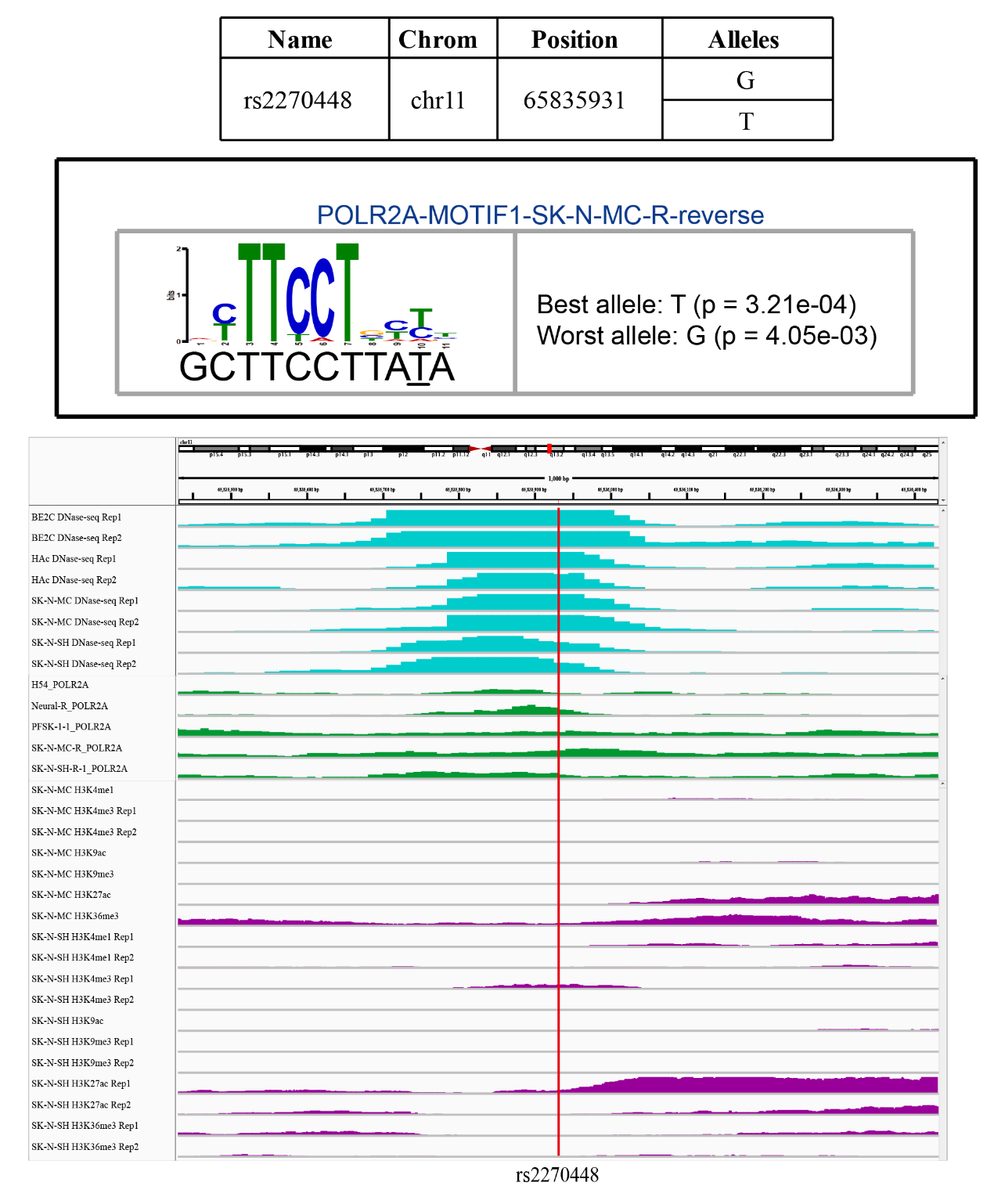

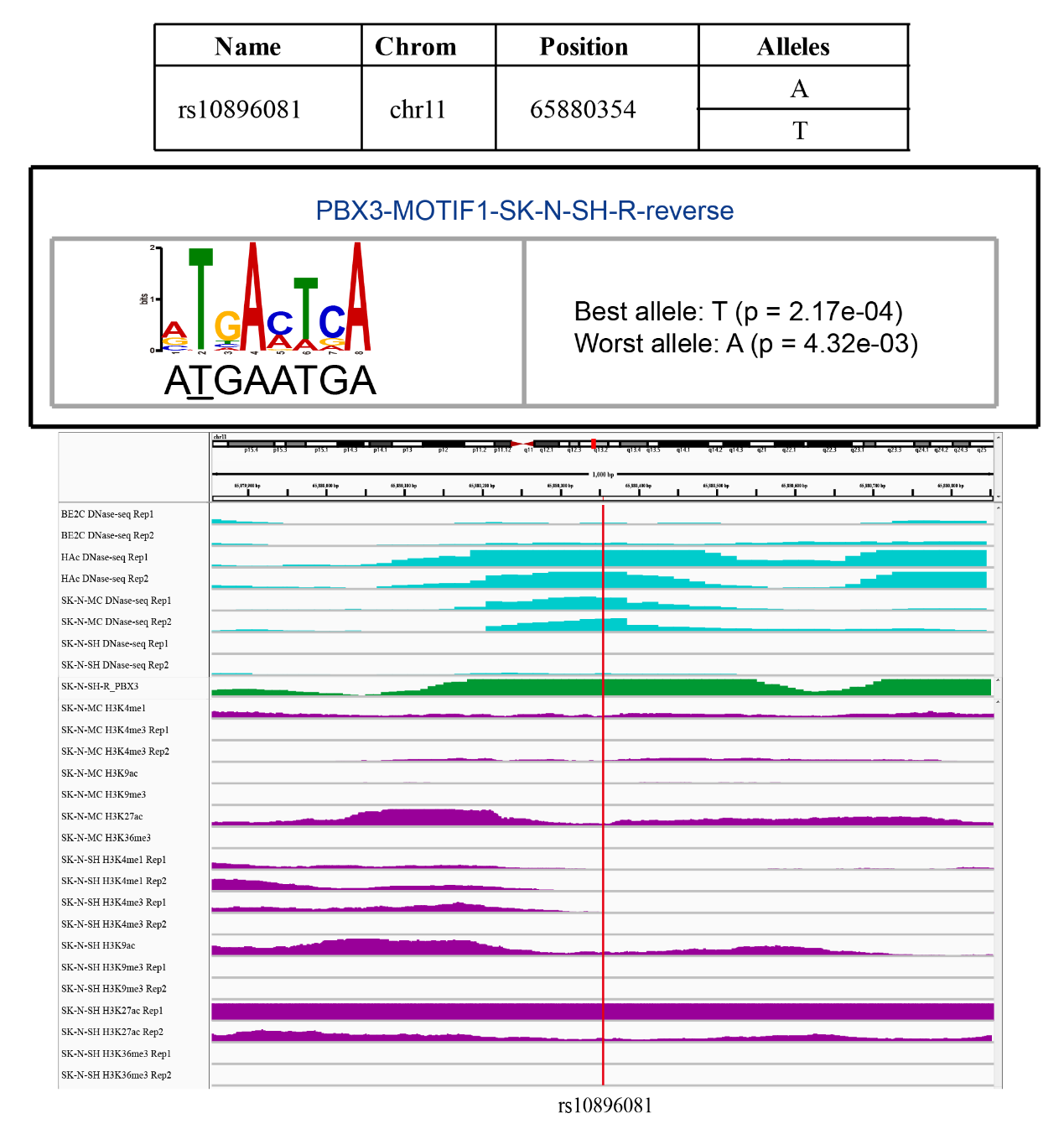

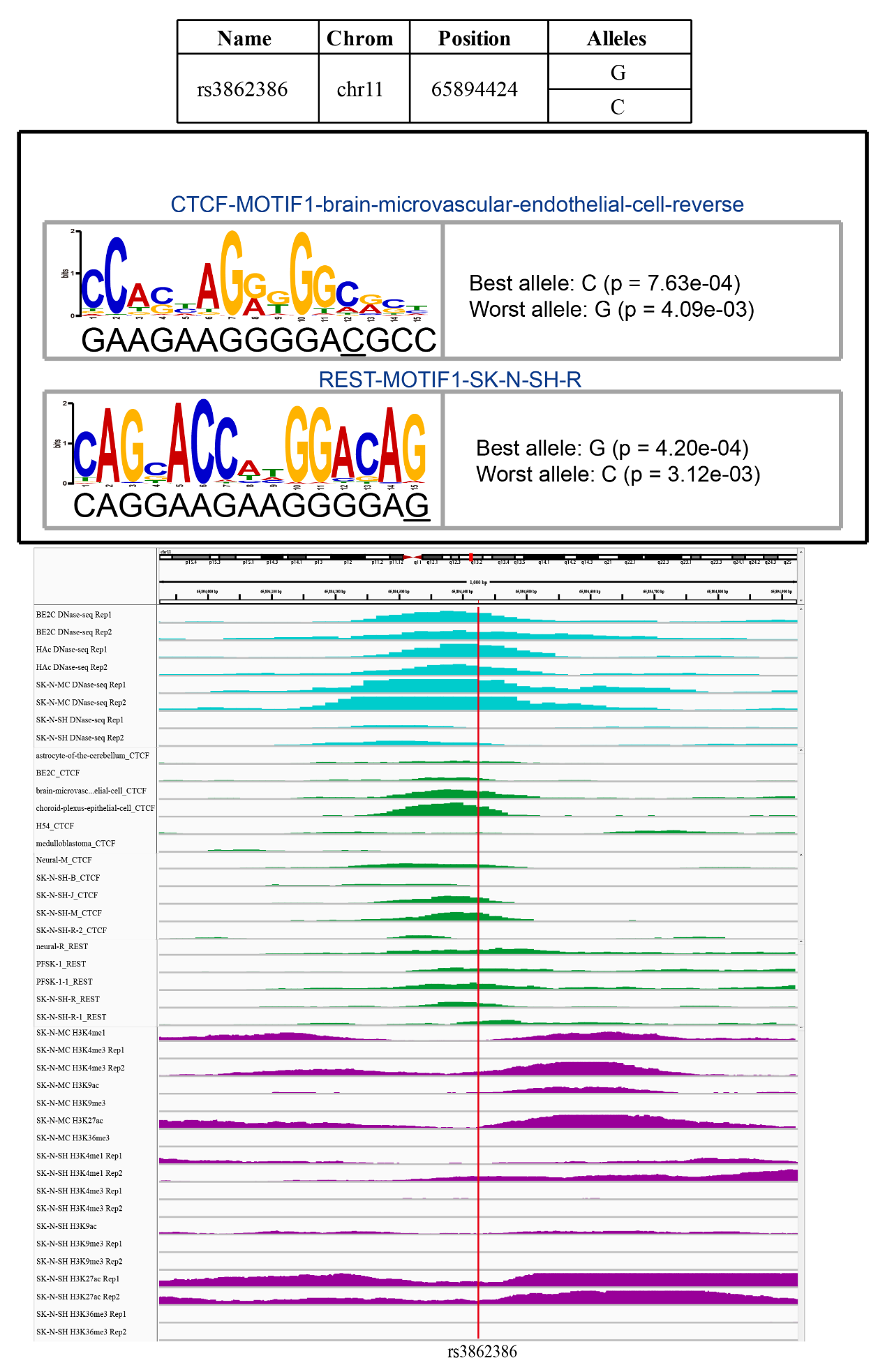

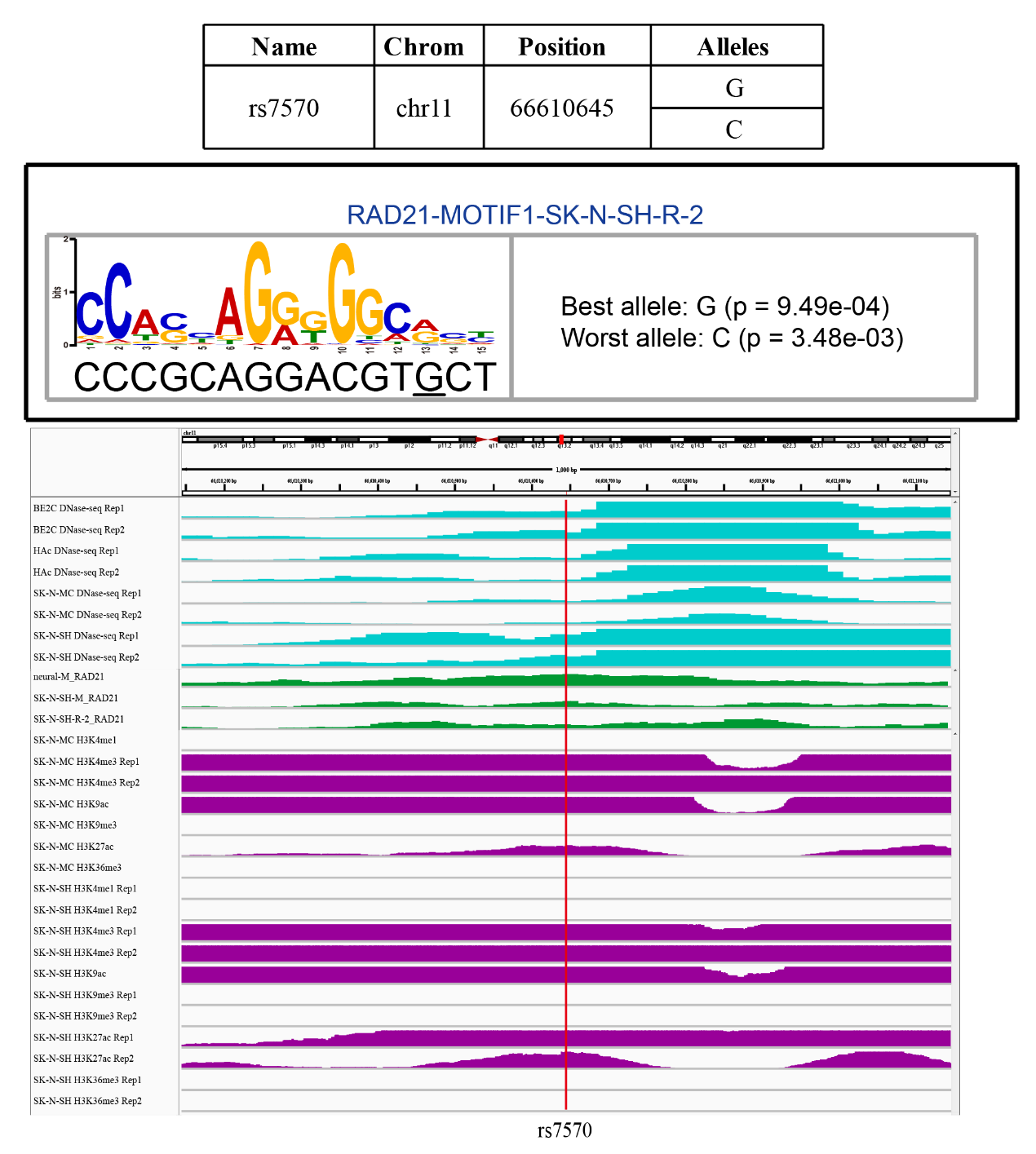

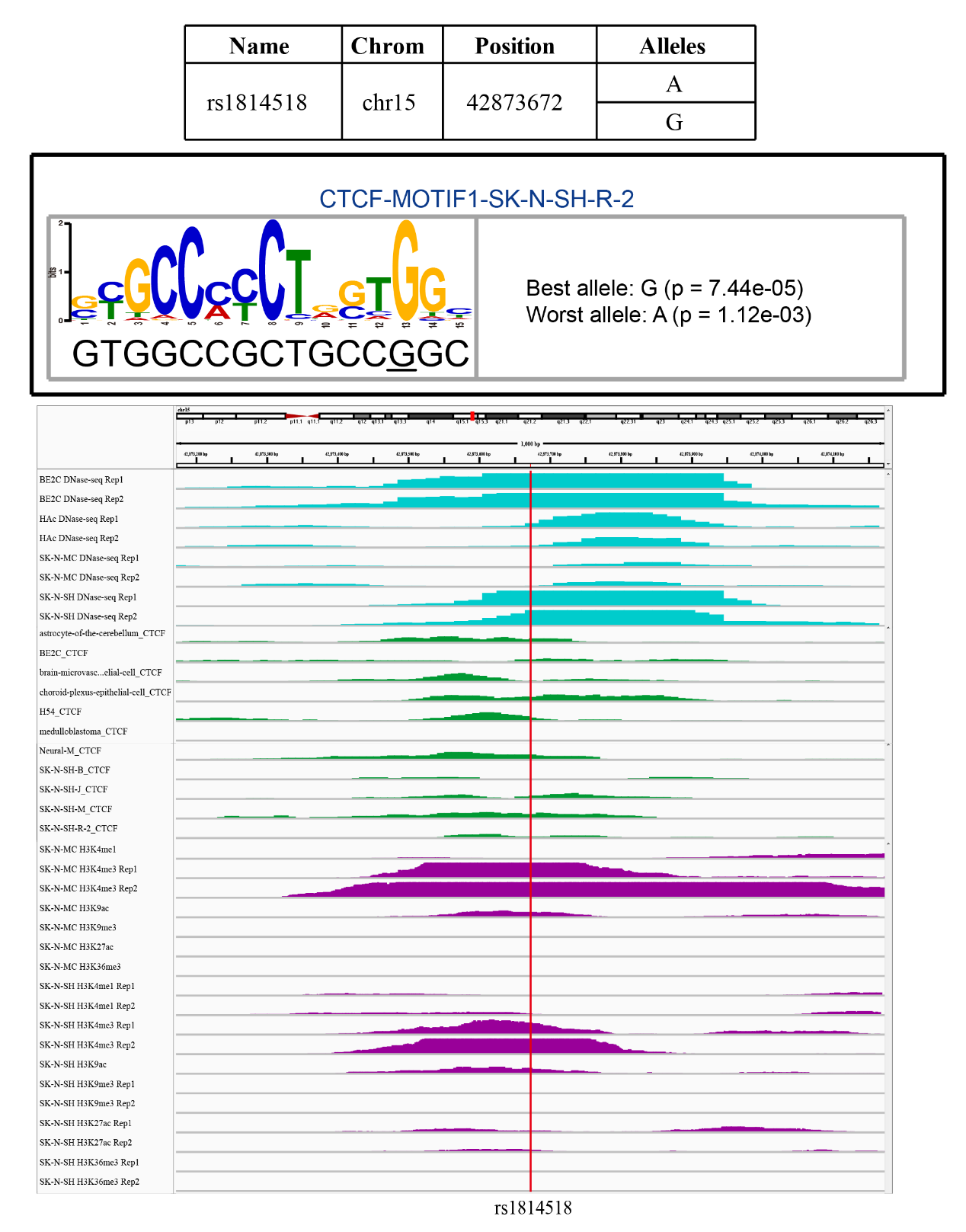

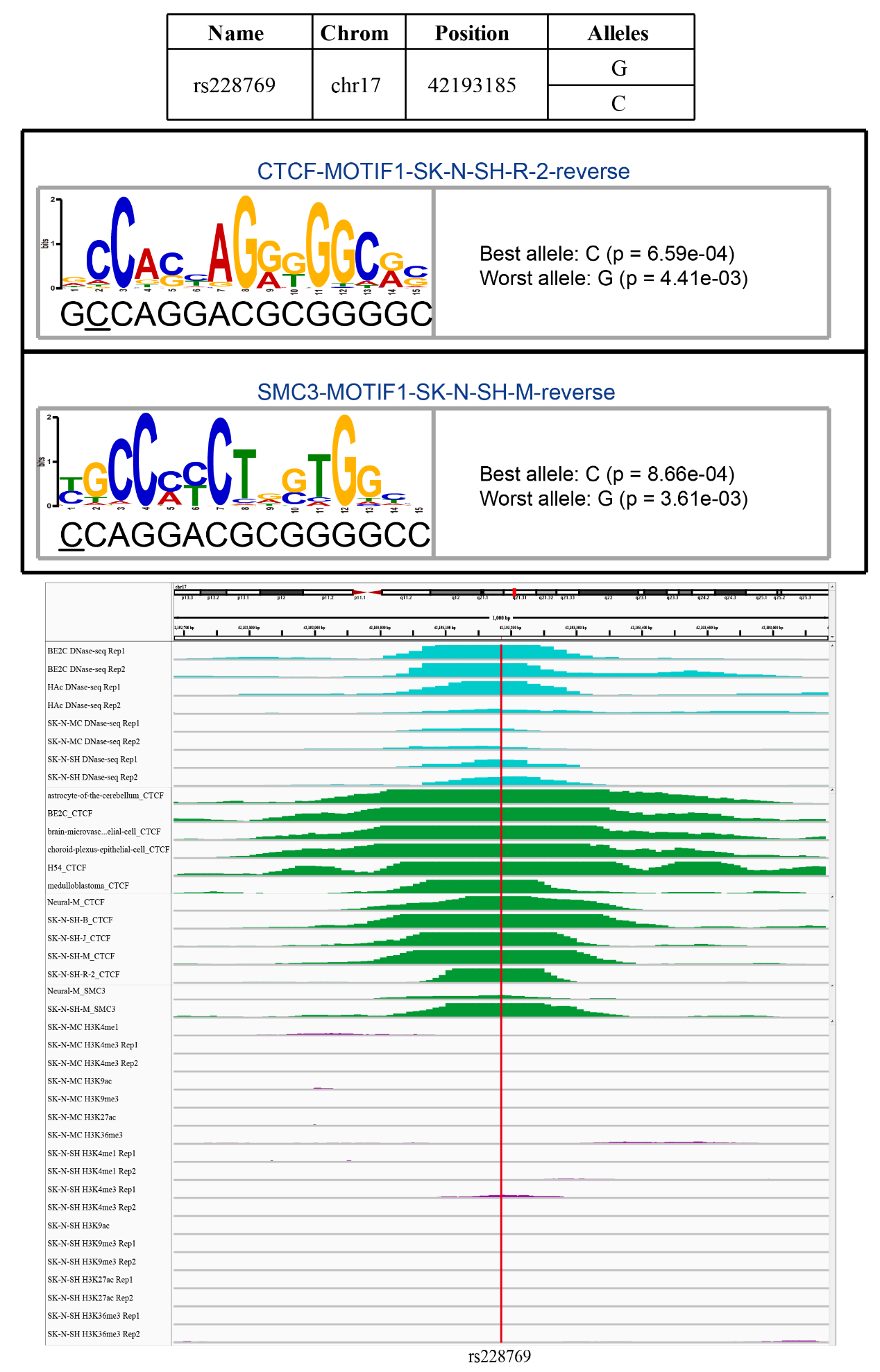

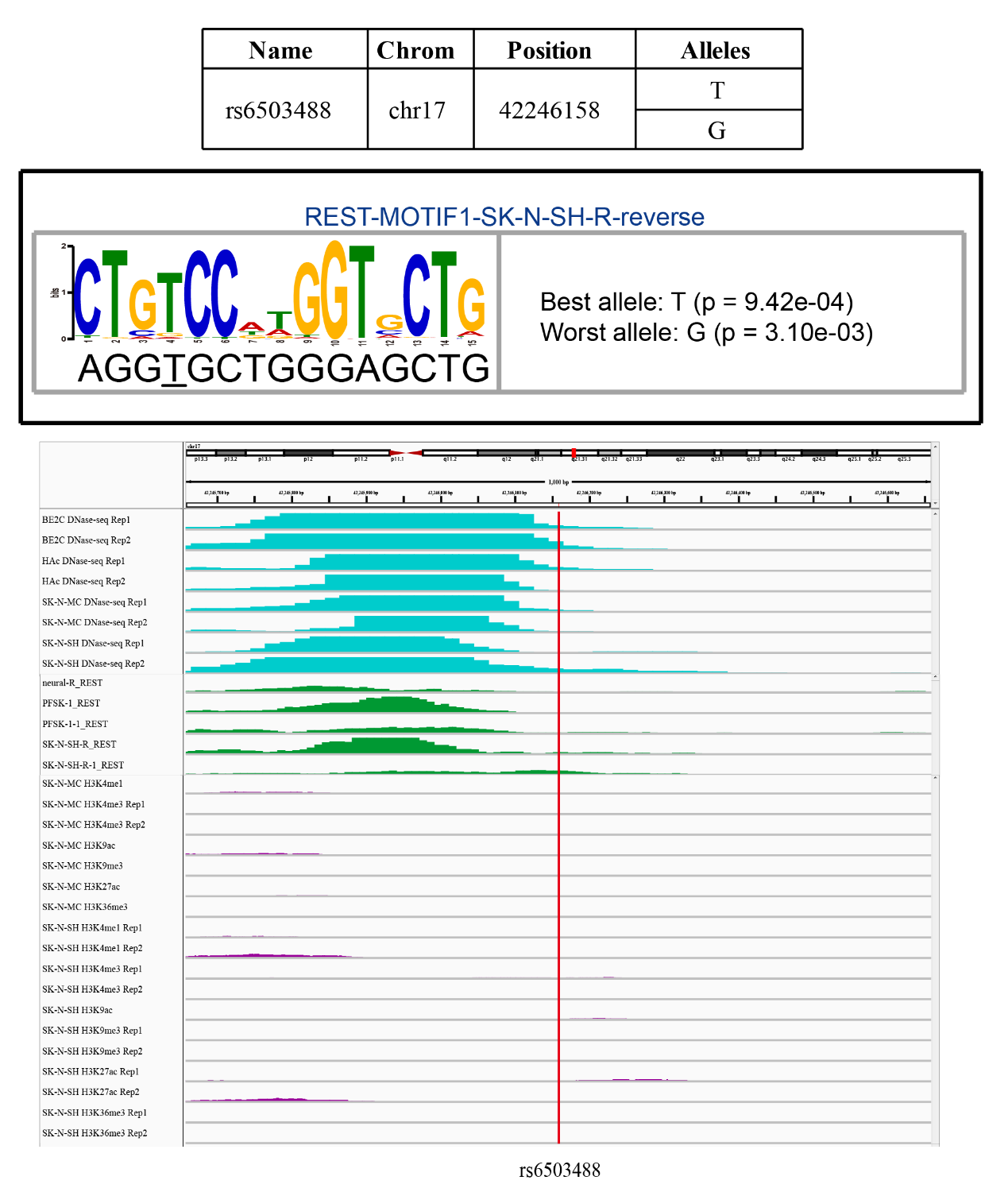

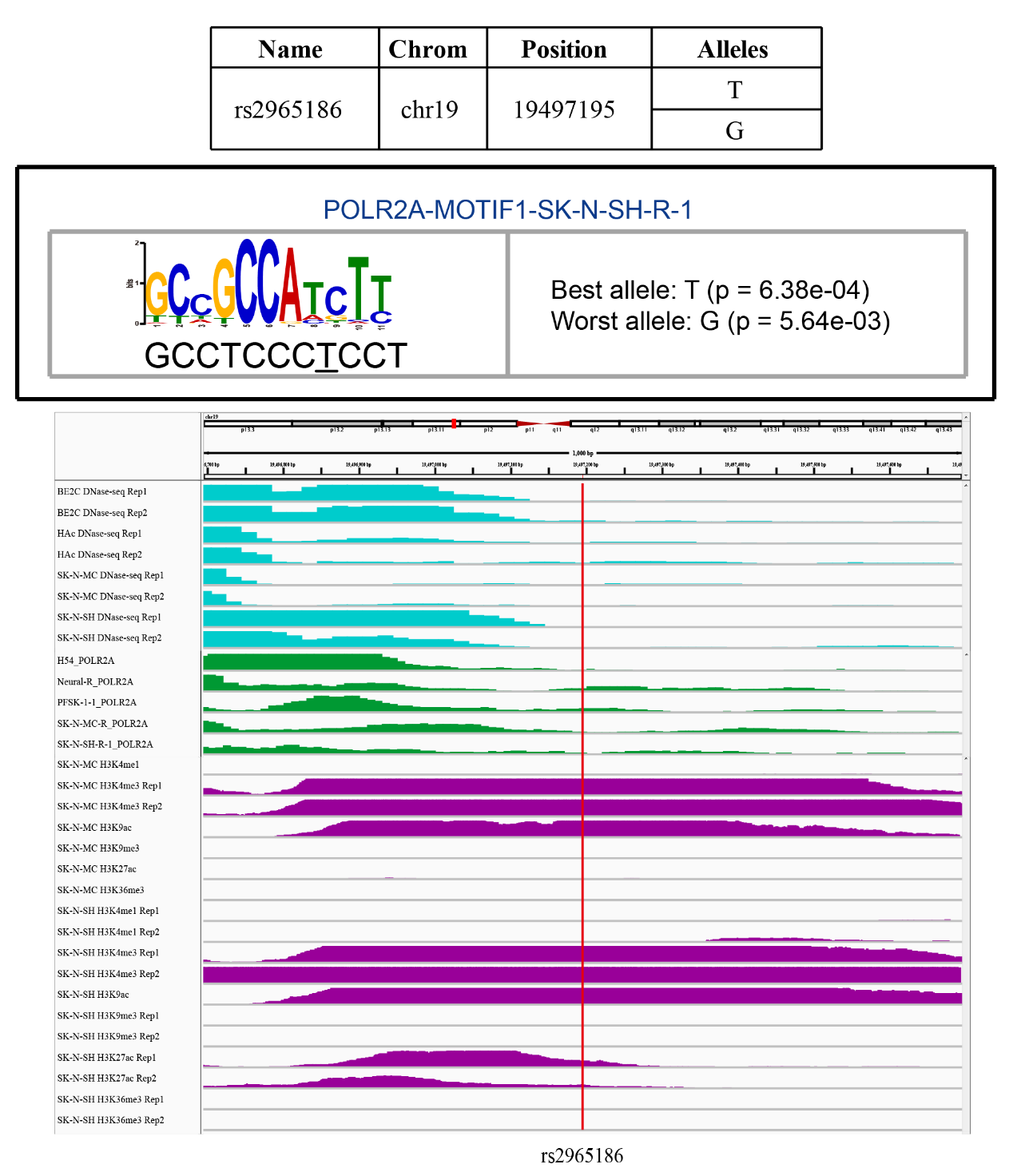

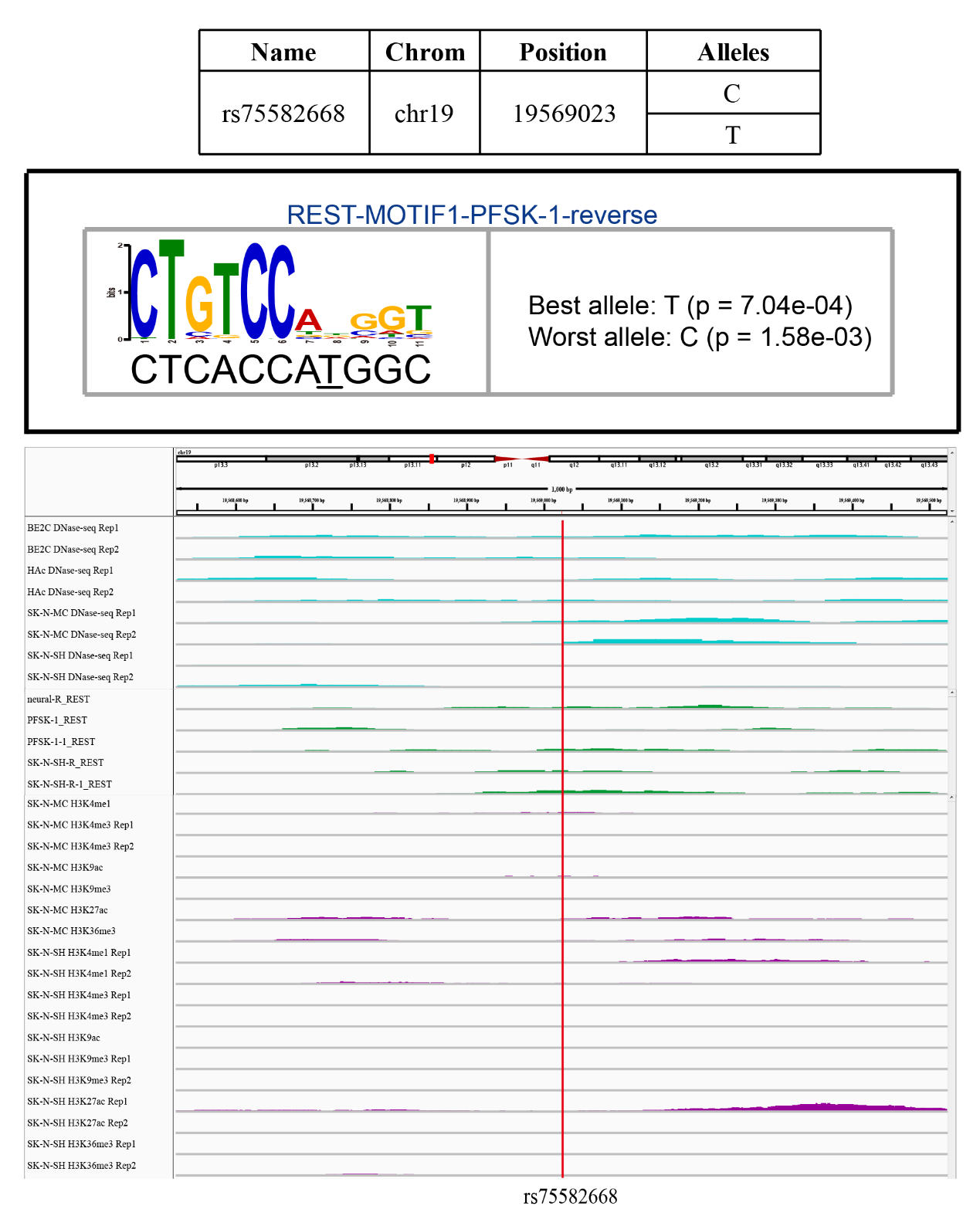

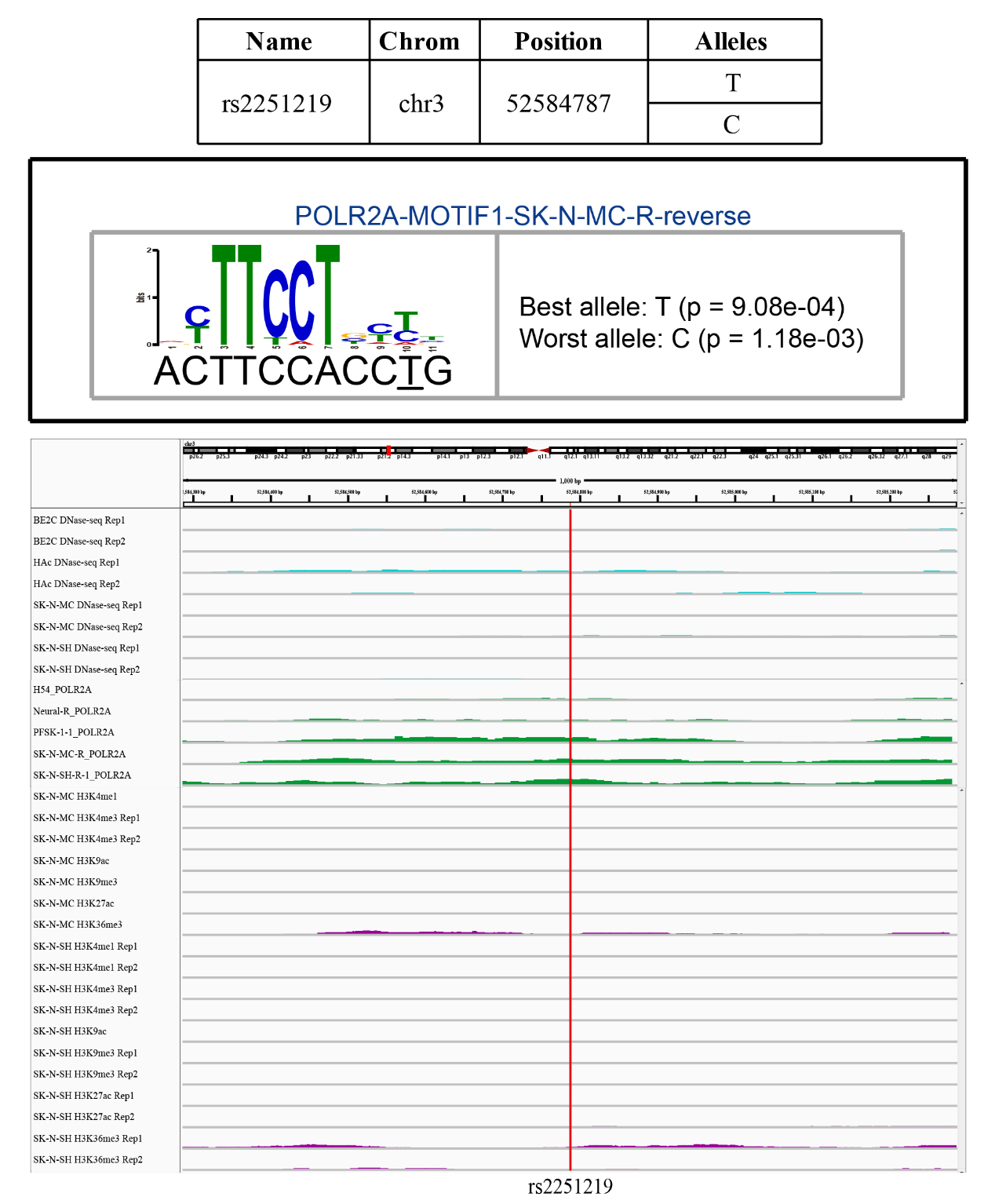

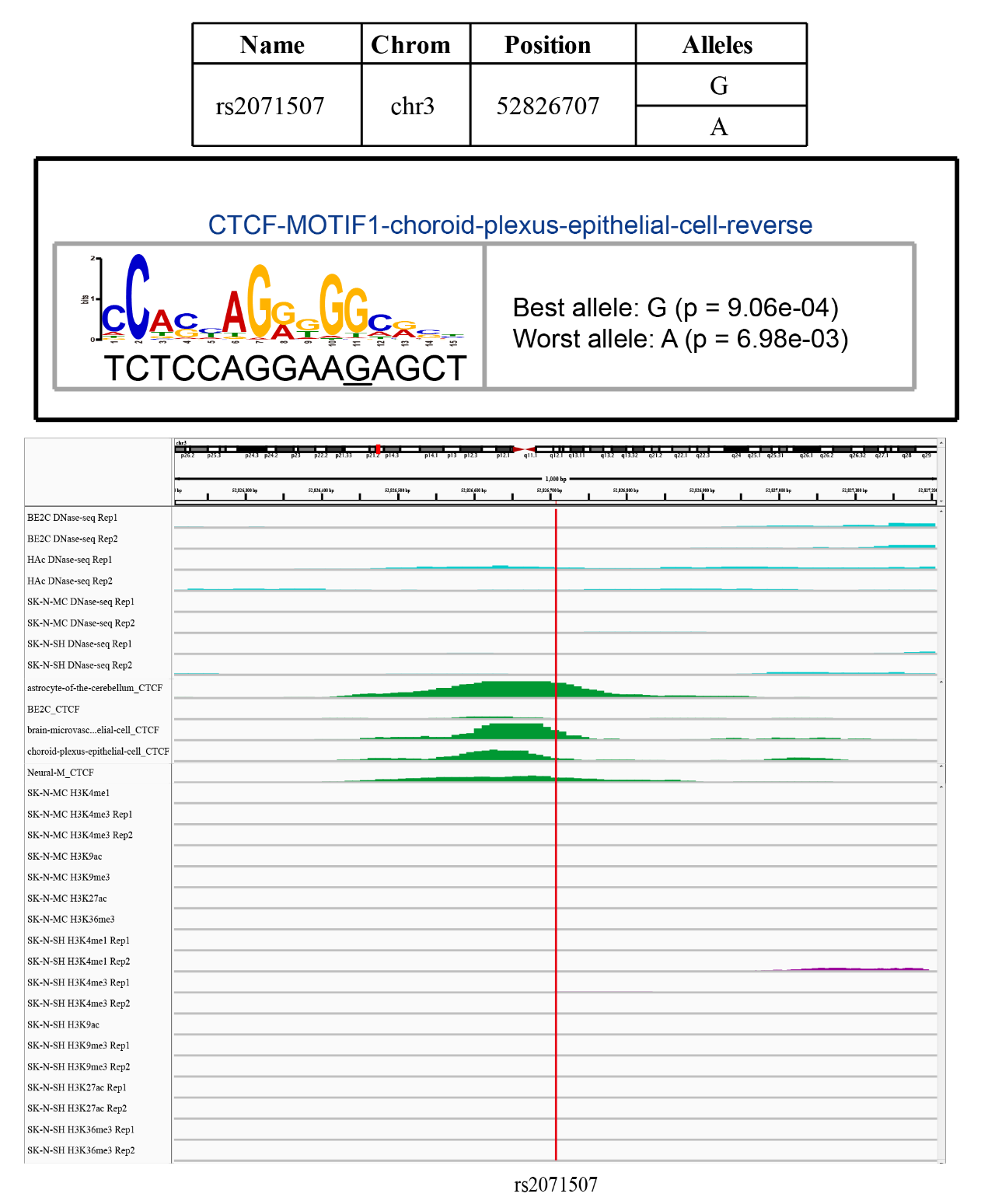

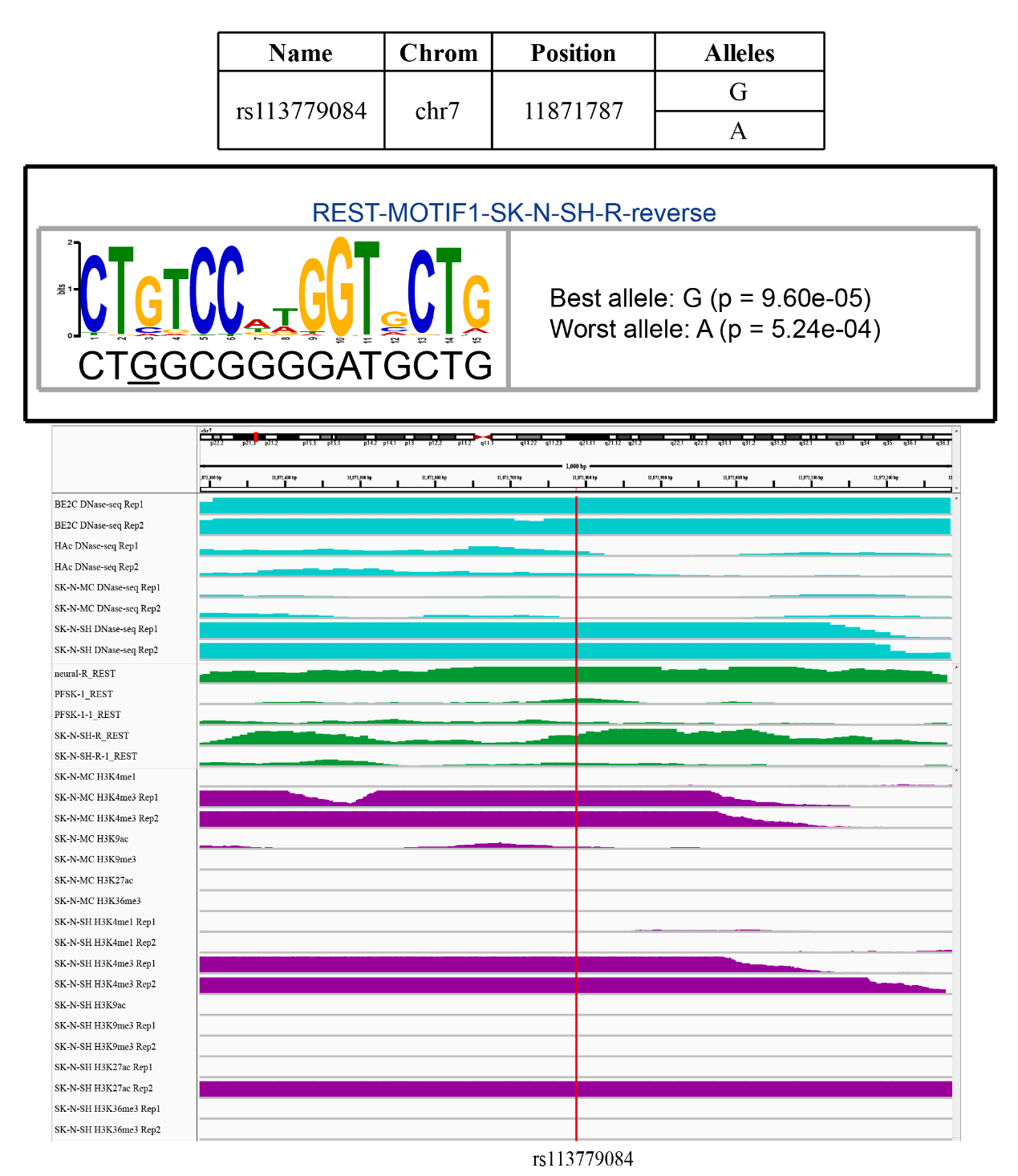


**Figure S5. The PWM data, ChIP-Seq, DNase-Seq, and histone modification of the 16 TF binding disrupting SNPs.**

**References**

1. ENCODE Project Consortium. An integrated encyclopedia of DNA elements in the human genome. Nature. 2012;489(7414):57-74. Available from: <https://www.encodeproject.org/>.

2. Whitington T, Gao P, Song W, Ross-Adams H, Lamb AD, Yang Y, et al. Gene regulatory mechanisms underpinning prostate cancer susceptibility. Nat Genet. 2016;48(4):387-97. Available from: https://www.ncbi.nlm.nih.gov/geo/query/acc.cgi?acc=GSE70770.

3. Wang D, Liu S, Warrell J, Won H, Shi X, Navarro FCP, et al. Comprehensive functional genomic resource and integrative model for the human brain. Science. 2018;362(6420): eaat8464. Available from: <http://resource.psychencode.org/>.

4. Collado-Torres L, Burke EE, Peterson A, Shin J, Straub RE, Rajpurohit A, et al. Regional Heterogeneity in Gene Expression, Regulation, and Coherence in the Frontal Cortex and Hippocampus across Development and Schizophrenia. Neuron. 2019;103(2):203-16. Available from: <http://eqtl.brainseq.org/phase2/eqtl/>.

5. Ng B, White CC, Klein HU, Sieberts SK, McCabe C, Patrick E, et al. An xQTL map integrates the genetic architecture of the human brain's transcriptome and epigenome. Nature neurosci. 2017;20(10):1418-26. Available from: http://mostafavilab.stat.ubc.ca/xQTLServe/.

6. Fromer M, Roussos P, Sieberts SK, Johnson JS, Kavanagh DH, Perumal TM, et al. Gene expression elucidates functional impact of polygenic risk for schizophrenia. Nature neurosci. 2016;19(11):1442-53. Available from: https://www.ncbi.nlm.nih.gov/geo/query/acc.cgi?acc=GSE30272.

7. GTEx Consortium: The GTEx Consortium atlas of genetic regulatory effects across human tissues. Science. 2020;369(6509):1318-30. Available from: <https://gtexportal.org/home/>.

8. Kong Y. Btrim: a fast, lightweight adapter and quality trimming program for next-generation sequencing technologies. Genomics. 2011;98(2):152-3. Available from: <http://graphics.med.yale.edu/trim/>.

9. Li H, Handsaker B, Wysoker A, Fennell T, Ruan J, Homer N, et al. The Sequence Alignment/Map format and SAMtools. Bioinformatics. 2009, 25(16):2078-9. Available from: [http://samtools.sourceforge.net](http://samtools.sourceforge.net/).

10. Langmead B, Trapnell C, Pop M, Salzberg SL. Ultrafast and memory-efficient alignment of short DNA sequences to the human genome. Genome biol. 2009;10(3):R25. Available from: <http://bowtie-bio.sourceforge.net/index.shtml>.

11. Zhang Y, Liu T, Meyer CA, Eeckhoute J, Johnson DS, Bernstein BE, et al. Model-based analysis of ChIP-Seq (MACS). Genome biol. 2008;9(9):R137. Available from: <http://liulab.dfci.harvard.edu/MACS/>.

12. Bailey TL, Elkan C. Fitting a mixture model by expectation maximization to discover motifs in biopolymers. Proc Int Conf Intell Syst Mol Biol. 1994;2:28-36. PMID: 7584402. Available from: <https://meme-suite.org/meme/tools/meme>.

13. Grant CE, Bailey TL, Noble WS. FIMO: scanning for occurrences of a given motif. Bioinformatics. 2011;27(7):1017-8. Available from: https://meme-suite.org/meme/tools/fimo.

14. Purcell S, Neale B, Todd-Brown K, Thomas L, Ferreira MA, Bender D, et al. PLINK: a tool set for whole-genome association and population-based linkage analyses. Am J Hum Genet. 2007;81(3):559-75. Available from: <http://zzz.bwh.harvard.edu/plink/>.
